# GRHL2 regulates keratinocyte EMT-MET dynamics and scar formation during cutaneous wound healing

**DOI:** 10.1038/s41419-024-07121-7

**Published:** 2024-10-14

**Authors:** Tianying Chen, Bo Zhang, Hanqi Xie, Chenyu Huang, Qiong Wu

**Affiliations:** 1 MOE Key Laboratory of Bioinformatics, Center for Synthetic and Systems Biology, School of Life Sciences, Tsinghua University, Beijing, 100084 China; 2 Department of Dermatology, Beijing Tsinghua Changgung Hospital, School of Clinical Medicine, Tsinghua University, Beijing, 102218 China

**Keywords:** Trauma, Cell biology

## Abstract

After cutaneous wounds successfully heal, keratinocytes that underwent the epithelial-mesenchymal transition (EMT) regain their epithelial characteristics, while in scar tissue, epidermal cells persist in a mesenchymal state. However, the regulatory mechanisms governing this reversion are poorly understood, and the impact of persistent mesenchymal-like epidermal cells in scar tissue remains unclear. In the present study, we found that during wound healing, the regulatory factor GRHL2 is highly expressed in normal epidermal cells, downregulated in EMT epidermal cells, and upregulated again during the process of mesenchymal-epithelial transition (MET). We further demonstrated that interfering with GRHL2 expression in epidermal cells can effectively induce the EMT. Conversely, the overexpression of GRHL2 in EMT epidermal cells resulted in partial reversion of the EMT to an epithelial state. To investigate the effects of failed MET in epidermal cells on skin wound healing, we interfered with GRHL2 expression in epidermal cells surrounding the cutaneous wound. The results demonstrated that the persistence of epidermal cells in the mesenchymal state promoted fibrosis in scar tissue, manifested by increased thickness of scar tissue, deposition of collagen and fibronectin, as well as the activation of myofibroblasts. Furthermore, the miR-200s/Zeb1 axis was perturbed in GRHL2 knockdown keratinocytes, and transfection with miR-200s analogs promoted the reversion of EMT in epidermal cells, which indicates that they mediate the EMT process in keratinocytes. These results suggest that restoration of the epithelial state in epidermal cells following the EMT is essential to wound healing, providing potential therapeutic targets for preventing scar formation.

## Introduction

Skin injuries are very common in daily life and mostly heal within weeks in healthy individuals. However, larger injuries or the presence of certain adverse factors—including aging, infection, diabetes, and vascular disease—can impede wound healing. These adverse factors result in the formation of chronic wounds or promote abnormal wound healing, leaving behind hypertrophic scars or more severe keloids [1–3]. Keratinocytes at the leading epidermal edge of a wound undergo the epithelial-mesenchymal transition (EMT), leading to a transition from cuboidal to flattened and elongated cell shapes, alterations in cell-cell adhesion and reorganization of the cytoskeleton [4, 5]. At the molecular level, there is a decrease in the expression of the epithelial marker gene E-cadherin, along with an increase of fibronectin and vimentin, which are mesenchymal markers [6–8]. Slug is involved in the regulation of the EMT in keratinocytes [9–11]. EMT is crucial for re-epithelialization, as evidenced by the delay of re-epithelialization in Slug-deficient mice [12]. During cutaneous wound healing, E-cadherin expression decreases three days after injury, and returns to normal levels within 1 or 2 days after re-epithelialization, indicating that the EMT is a dynamic, reversible process [13, 14]. Several studies have reported the activation of a mesenchymal-like phenotype in keratinocytes within keloids and hypertrophic scar tissue, suggesting that EMT keratinocytes may be involved in the formation of abnormal scar tissue [15–18]. Keloids are a special type of scar tissue that extends beyond the original wound boundaries and continues to enlarge persistently, without regressing over time. Moreover, the treatment of keloids with surgical excision or radiotherapy often increases the risk of recurrence [19, 20].

Nevertheless, we still incompletely understand the regulatory mechanisms orchestrating the transition of mesenchymal-like keratinocytes back to an epithelial state, which is termed the mesenchymal epithelial transition (MET). Additionally, the implications of persisting mesenchymal-like keratinocytes for scar remodeling remain uncertain.

Grainyhead-like 2 (GRHL2), a transcription factor, along with GRHL1 and GRHL3, are the three known mammalian homologs of the *Drosophila* Grainyhead (GRH) gene, playing key roles in epithelial morphogenesis [21, 22]. In mammalian keratinocytes, GRHL2 regulates cell proliferation [23] and terminal differentiation [24]. Moreover, it has been widely described as an EMT suppressor in various cancer cell types, including breast cancer [25–27], ovarian cancer [28], and gastric cancer [29]. In general, GRHL2 suppresses the EMT and is downregulated in disseminated cancer cells in the mesenchymal state. Conversely, GRHL2 overexpression leads to MET. In addition, embryos with heterozygous deletion of GRHL2 exhibit defects in limb amputation healing [30]. The results of our study show that the compromised epithelialization of mesenchymal-like keratinocytes regulated by GRHL2 leads to fibrosis of the scar tissues after wounds have healed.

## Results

### GRHL2 is downregulated in epidermal cells undergoing the EMT during cutaneous wound healing

**Fig. 1:**
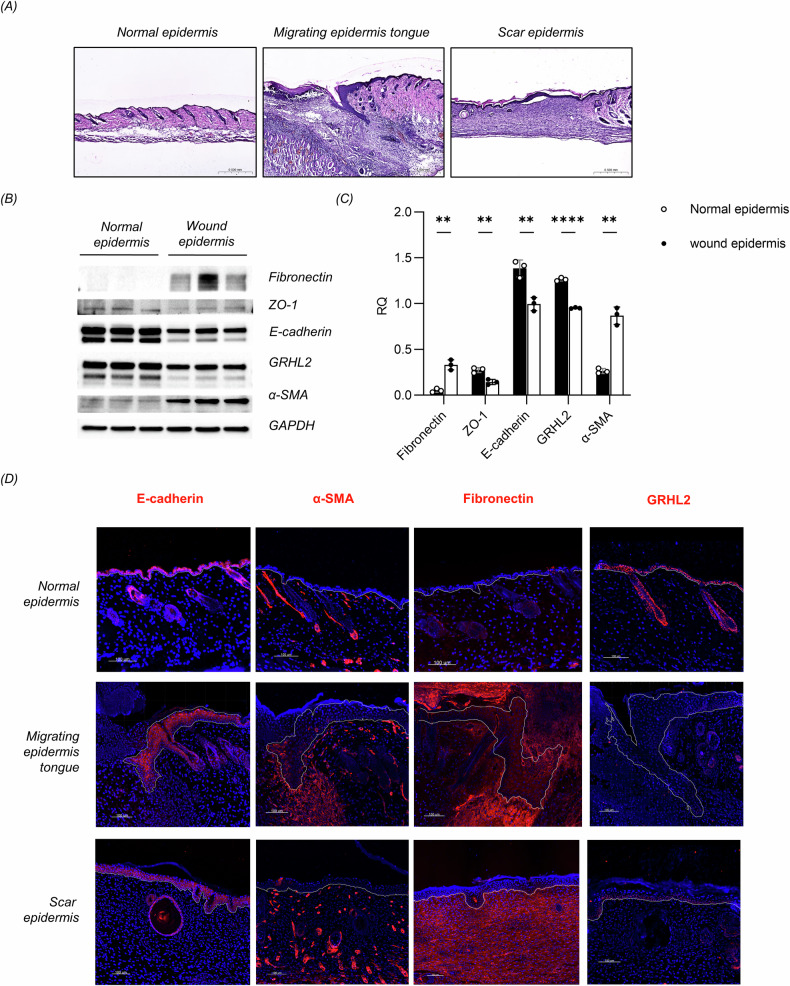
Changes of GRHL2 expression in epidermal cells during wound healing. **A** HE staining of mouse skin sections from different stages of cutaneous wound healing. Scale bar = 500 μm. **B** The total protein extracted from the epidermis at the wound edge and normal epidermis was analyzed for fibronectin, ZO-1, E-cadherin, GRHL2 and α-SMA by western blot. **C** Quantification of protein bands from the western blot in Fig. 1B. **D** Immunofluorescence analysis of E-cadherin, α-SMA, fibronectin and GRHL2 expression in the epidermis during cutaneous wound healing. Blue indicates cell nuclei. White dotted lines indicate the epidermis determined by immunofluorescence of keratin 14. Scale bar = 100 μm.

To investigate the mechanism by which EMT keratinocytes restore their epithelial state during wound healing, we focused on the MET-regulated factor GRHL2, which has been implicated in tumor cells. Full-thickness wounds extending through the panniculus carnosus with a diameter of 1 cm were created on the dorsum, and wound closure was observed to occur within approximately 14 days (Supplementary Fig. 1A). Epidermal cells at the edge of the wound formed an epidermal migratory tongue (Fig. 1A). Epidermis samples were collected from the area around the wound, and western blot analysis revealed EMT in the epidermal tissue, characterized by the downregulation of epithelial markers such as E-cadherin and ZO-1, as well as the upregulation of mesenchymal markers such as α-SMA and fibronectin. Notably, GRHL2 was downregulated in epidermal cells undergoing the EMT (Fig. 1B, C).

At the same time, immunofluorescence staining was performed to observe the dynamic transition of epidermal cells between epithelial and mesenchymal states during wound healing. Cells at the leading edge of the epidermis expressed the basal keratinocyte marker keratin 14. Accordingly, we determined the location of the migrating epidermal front by keratin 14 staining. The results demonstrated the activation of fibronectin and α-SMA, as well as the loss of E-cadherin in the epidermal migratory tongue, indicating EMT in epidermal cells. This EMT process was associated with a decrease of GRHL2 expression in keratinocytes. Following wound closure, GRHL2 expression was restored, coinciding with the epidermal cells regaining their epithelial phenotype (Fig. 1D, Supplementary Fig. 1B). We also examined surgically excised keloid samples and observed EMT in the epidermal component of keloids, along with a downregulation of GRHL2 expression (Supplementary Fig. 1C, D). These results offer strong evidence that there is a dynamic and reversible transition of epidermal cells between epithelial and mesenchymal states during wound healing, which is accompanied by dynamic changes of GRHL2 expression.

### Oscillatory changes of GRHL2 expression during EMT-MET dynamics in human keratinocytes

**Fig. 2:**
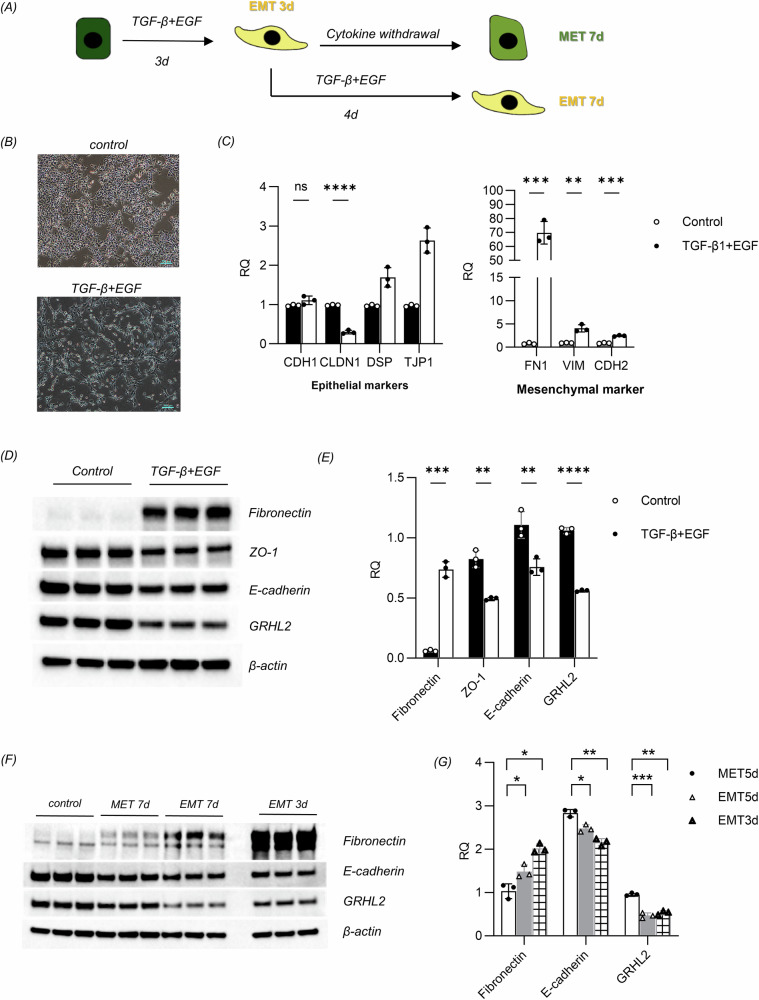
GRHL2 is upregulated in MET keratinocytes. **A** Schematic diagram of the construction of the dynamic EMT-MET model in human keratinocytes. **B** Morphological changes of keratinocytes undergoing the EMT. Scale bar = 100 μm. **C** Expression levels of the epithelial and mesenchymal markers in the control group as well as the group treated with TGF-β and EGF according to RT-qPCR. The data are presented as means ± SD, *p < 0.05, **p < 0.01, ***p < 0.001, ****p < 0.0001, *n* = 3. **D** Expression levels of EMT-related markers in control group as well as keratinocytes treated with TGF-β and EGF according to western blotting for fibronectin, ZO1, E-cadherin and GRHL2. **E** Quantification of protein bands from the western blot in **D**. **F** Expression levels of EMT-related markers in keratinocytes in the control, EMT and MET groups according to western blotting for E-cadherin and fibronectin. **G** Quantification of protein bands from the western blot in **F**, excluding the control group data. CDH1 E-cadherin, CLDN1 claudin 1, DSP desmoplakin, TJP1 ZO-1, FN1 fibronectin, VIM vimentin, CDH2 N-cadherin.

We further developed a model to simulate the dynamic transition of epidermal cells between the epithelial and mesenchymal states. In this model, we utilized TGF-β and EGF to induce the EMT in the immortalized human HaCaT keratinocyte cell line, followed by the withdrawal of these cytokines, allowing the cells to regain epithelial characteristics (Fig. 2A). Under TGF-β and EGF induction, the keratinocytes were observed to transition from a cuboidal morphology to a spindle-shaped appearance resembling fibroblasts, which is typical of mesenchymal cells (Fig. 2B).

RT-qPCR analysis revealed a significant upregulation of mesenchymal marker genes such as fibronectin (FN1), vimentin (VIM), and N-cadherin (CDH2), as well as the downregulation of claudin 1 (CLDN1), an epithelial marker gene (Fig. 2C). Additionally, western blot analysis revealed a decrease in the protein levels of the epithelial markers E-cadherin and ZO-1, along with the activation of fibronectin expression. A decrease of GRHL2 protein levels could also be observed in EMT keratinocytes (Fig. 2D, E).

After withdrawal of cytokines, RT-qPCR **(**Supplementary Fig. 2) and western blot (Fig. 2F, G) analyses revealed that some of the keratinocytes in the mesenchymal state reverted to the epithelial state. During this process, the expression levels of GRHL2 rebound, suggesting that oscillatory changes of GRHL2 expression levels are correlated with the EMT-MET dynamics of epidermal cells.

### Interfering with GRHL2 expression in epidermal cells induces the EMT

**Fig. 3:**
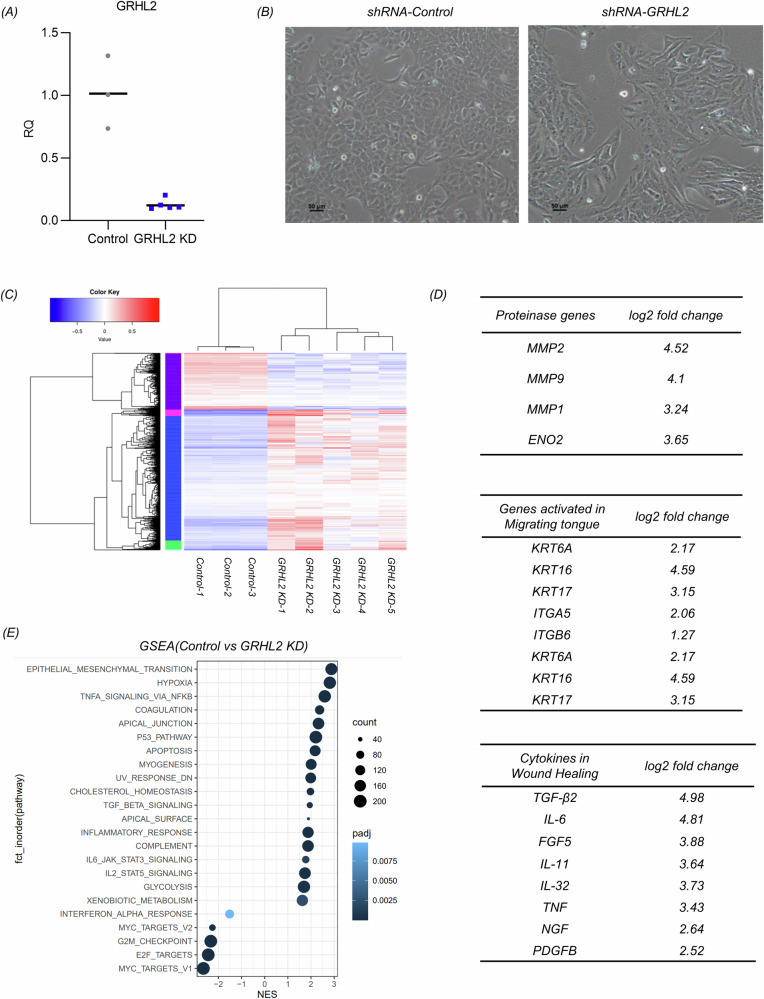
Transcriptomic analysis of the effects of GRHL2 knockdown in keratinocytes. **A** The knockdown efficiency of GRHL2 in keratinocytes according to RT-qPCR. Control: Double-stranded small RNAs that do not target any known mammalian genes. **B** Morphological changes of keratinocytes with GRHL2 knockdown. Scale bar = 50 μm. **C** Cluster analysis of transcriptomic datasets. **D** Transcriptomic analysis of differential gene expression (GRHL2 KD cells compared with control keratinocytes). **E** Gene set enrichment analysis (GSEA) of keratinocytes with GRHL2 knockdown compared to the control group.

To further confirm its role, we interfered with GRHL2 expression by delivering shRNA into HaCaT cells using a lentiviral vector. As the proliferative capacity of GRHL2 knockdown cells (GRHL2 KD) decreased, we established a relatively stable population of cells with low GRHL2 expression through monoclonal amplification (Fig. 3A). Microscopic observations revealed that keratinocytes with GRHL2 knockdown exhibited a spindle-shaped morphology (Fig. 3B). Transcriptomic analysis unveiled significant differences between GRHL2 KD keratinocytes and the control group (Fig. 3C). Differentially expressed genes included matrix metalloproteinase (MMP), keratins, and integrins, which were found to be activated at the leading epidermal edge, suggesting a potential association of GRHL2 inhibition with the formation of the epidermal migration front [31–35]. Moreover, we observed an increase in the expression levels of some scarring-related factors such as IL-6, suggesting that the persistence of EMT epidermal cells may promote scar tissue fibrosis (Fig. 3D) [36, 37]. The RT-qPCR results validated these gene expression changes in response to GRHL2 knockdown (Supplementary Fig. 3A, B). The increased expression of MMP2 suggested that GRHL2 knockdown may regulate the expression of E-cadherin, ZO-1, and related factors through post-transcriptional regulation (Supplementary Fig. 3C, D).

Gene set enrichment analysis (GSEA) highlighted a significant impact of GRHL2 knockdown on the EMT pathway, as well as noticeable effects on the apical junction and surface pathways. In addition, GSEA analysis also showed that GRHL2 knockdown also influenced the TGF-β, TNF-NFκB, and IL-6 signaling pathways in epidermal cells (Fig. 3E). Notably, these pathways are implicated in the formation of scars [38].

Cells undergoing the EMT typically exhibit reduced proliferation and enhanced migratory capacity [39–41]. The proliferation of GRHL2 KD and control cells was assessed using the Cell Counting Kit-8 (CCK-8). The kit contains a water-soluble tetrazolium salt that produces an orange formazan dye upon bio-reduction by cellular dehydrogenases. The amount of generated formazan is directly proportional to the number of living cells and is determined by measuring the absorbance at 460 nm. When inoculated at the same density, the proliferation capacity of GRHL2 KD cells was lower than that of control cells (Supplementary Fig. 3E). Dominguez et al. reported a high-resolution cell-cycle transcriptome map, which identified 1186 periodic mRNAs in different cell types [42]. After mapping these 1186 cell-cycle-related genes onto the transcriptome of GRHL2 knockdown keratinocytes (Supplementary Fig. 3F), we observed that G2-M phase related genes were significantly upregulated, especially carboxypeptidase A4 (CPA4) and amelotin (AMTN).

**Fig. 4:**
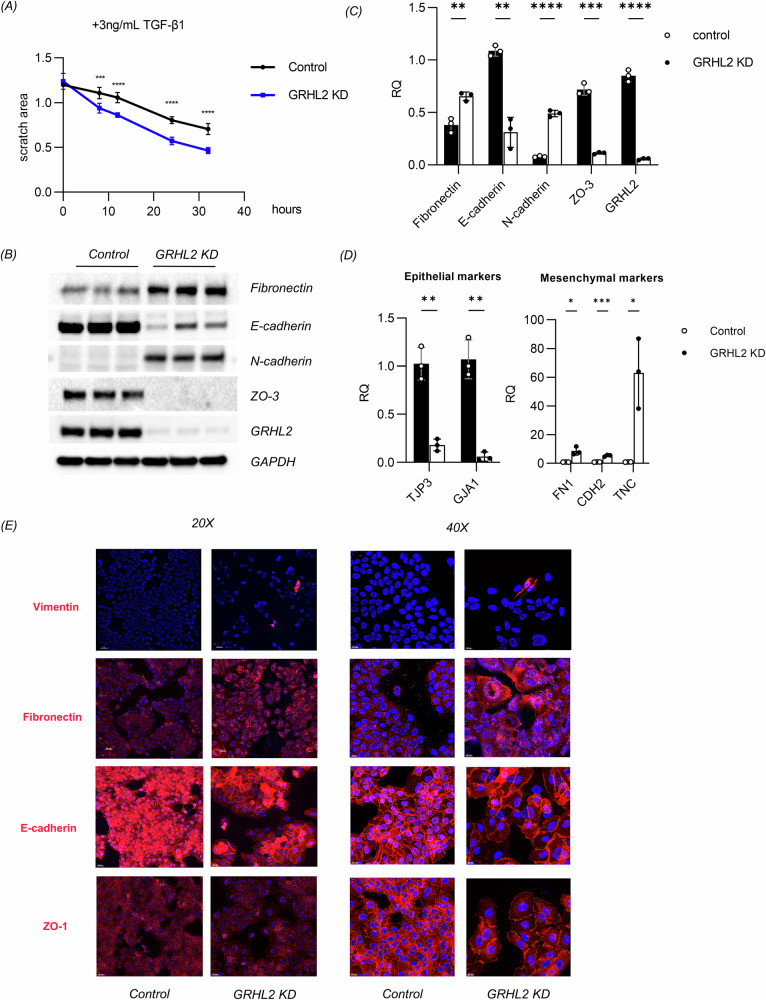
Knockdown of GRHL2 effectively induced the transition of epithelial cells into a mesenchymal state. **A** Wound healing assay to determine cell migration. Photos of the scratch for analysis were taken at 0 h, 8 h, 12 h, 24 h and 32 h in monolayers of control and GRHL2 KD keratinocytes, respectively. The remaining scratch area was determined based on microscopic images using ImageJ. The data are presented as means ± SD, *p < 0.05, **p < 0.01, ***p < 0.001, ****p < 0.0001, *n* = 6. **B** Expression levels of EMT-related markers in normal and GRHL2 KD keratinocytes according to western blotting for fibronectin, E-cadherin, N-cadherin, ZO-3 and GRHL2. **C** Quantification of protein bands from the western blot in **B**. **D** Expression levels of epithelial and mesenchymal genes according to RT-qPCR in control and GRHL2 KD keratinocytes. The data are presented as means ± SD, *n* = 3. *p < 0.05, **p < 0.01, ***p < 0.001, ****p < 0.0001. **E** Immunofluorescence analysis of vimentin, fibronectin, E-cadherin and ZO-1 in control and GRHL2 KD keratinocytes. Blue indicates the cell nuclei. Scale bar = 40 μm (20×) or 20μm (40×). TJP3 (ZO-3), GJA1 (gap junction protein alpha 1), FN1 (fibronectin), CDH2 (N-cadherin), TNC (tenascin-C).

The wound healing assay was utilized to evaluate the migratory capacity of the cells. To mimic epidermal cell migration during wound healing, we added a low concentration of TGF-β (3 ng/mL) to the culture medium. The experimental findings revealed that the migration rate of GRHL2 KD cells was significantly higher than that of control cells (Fig. 4A). In addition, western blot (Fig. 4B, C), qPCR (Fig. 4D) and immunofluorescence staining (Fig. 4E, Supplementary fig. 3G) were employed to further assess the changes in the expression of EMT-related markers. The experimental results showed that interfering with GRHL2 expression in keratinocytes induced the EMT. The RT-qPCR results showed that the inhibition of GRHL2 reduced the mRNA expression of cell adhesion molecule ZO-3 (TJP3) and gap junction protein alpha 1 (GJA1), while upregulating FN1, CDH2 and tenascin-C (TNC). The expression of these genes is activated during the EMT. The western blot analysis also verified the change of EMT-related markers in GRHL2 KD keratinocytes. According to the immunofluorescence staining, we noticed that some cells lost the expression of E-cadherin and ZO-1, predominantly located at the edge of the cell islands. This suggests a regulatory role of intercellular contacts in the EMT. Although activation of vimentin expression was exceedingly rare and was not shown in the differential gene expression list, there was nevertheless a discernible difference between the experimental and control groups. The qPCR results also demonstrated that GRHL2 knockdown can increase the transcription level of the vimentin gene (VIM) (Supplementary Fig. 3H).

In summary, GRHL2 knockdown effectively induced the EMT in keratinocytes. Therefore, interference with GRHL2 expression in the epidermis surrounding wounds could prevent the MET of keratinocytes. Transcriptomic analysis further indicated that the downregulation of GRHL2 expression is associated with phenotypic changes in cells at the migrating epidermal tongue, while EMT keratinocytes with low GRHL2 expression may affect scar remodeling by enhancing the secretion of cytokines such as IL-6 and MMPs.

### GRHL2 overexpression promoted the MET in Keratinocytes

**Fig. 5:**
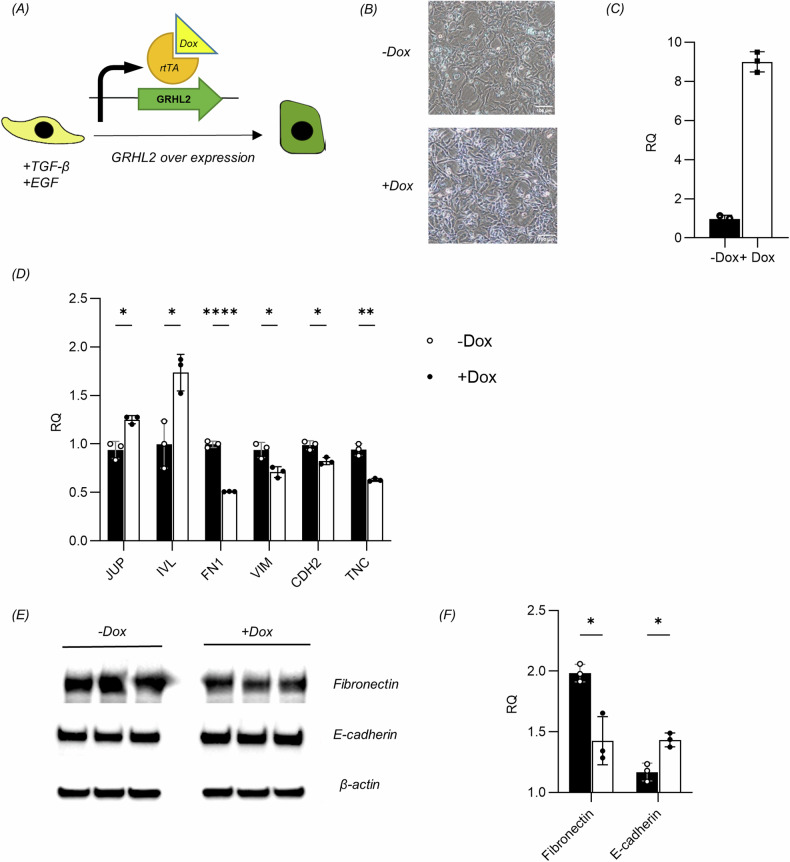
GRHL2 overexpression promoted the MET of Keratinocytes. **A** Schematic of doxycycline (Dox)-inducible overexpression of GRHL2 in EMT keratinocytes. **B** Morphological changes of EMT keratinocytes following GRHL2 overexpression. **C** Overexpression efficiency of GRHL2 in EMT keratinocytes according to RT-qPCR. **D** Expression levels of EMT-related markers according to RT-qPCR in EMT keratinocytes treated with Dox or left untreated. Error bars represent standard deviations, *n* = 3. *p < 0.05, **p < 0.01, ***p < 0.001, ****p < 0.0001. JUP (junction plakoglobin), IVL (involucrin), FN1 (fibronectin), VIM (vimentin), CDH2 (N-cadherin), TNC (tenascin-C). **E** Expression levels of fibronectin and E-cadherin according to western blot analysis in EMT keratinocytes treated with Dox or left untreated. **F** Quantification of protein bands from the western blot in **E**.

After investigating the effects of gene knockdown, we attempted to overexpress GRHL2 to see if it would promote the MET of keratinocytes. To achieve this, we constructed a cassette for doxycycline (Dox)-induced expression of GRHL2 and introduced it into the HaCaT keratinocyte cell line on a lentiviral vector (Fig. 5A).

After inducing the EMT, the culture medium was supplemented with 0.4 mg/L Dox, while maintaining the presence of TGF-β and EGF. The transcriptional level of GRHL2 substantially increased in response to Dox, as evidenced by the qPCR results (Supplementary Fig. 4A). We then detected the changes of EMT-related markers following Dox induction using qPCR. Our findings (Supplementary Fig. 4B) demonstrated that GRHL2 overexpression failed to raise the expression of CDH1, TJP1, and CLDN1. Nevertheless, it led to an upregulation of the genes encoding involucrin (IVL) and junction plakoglobin (JUP) after 48 hours, accompanied by a decrease in the expression of the mesenchymal genes FN1, VIM, and CDH2. However, western blot analysis (Supplementary Fig. 4C, D) indicated that GRHL2 overexpression had a limited impact on reversing the EMT phenotype of keratinocytes. Since induction with TGF-β and EGF can substantially reduce the protein expression level of GRHL2, but has little effect on the amount of GRHL2 mRNA, this treatment likely inhibits translation, thus impeding the ability of GRHL2 overexpression to reverse the EMT phenotype.

Therefore, we proposed to induce GRHL2 overexpression after withdrawing TGF-β and EGF from the culture medium. After cytokine withdrawal and supplementation of Dox for 48 hours, a decrease in the percentage of spindle-shaped cells could be observed (Fig. 5B). Consistent with previous results, Dox induction markedly increased the transcript levels of GRHL2 (Fig. 5C). In addition, we also examined the transcription levels of EMT markers by qPCR (Fig. 5D). The epithelial genes JUP and IVL were upregulated after 48 h of Dox induction, while FN1, CDH2, and TNC showed a concomitant decrease. Furthermore, Dox induction downregulated the protein expression of fibronectin, while the protein level of E-cadherin significantly rebounded (Fig. 5E, F).

Although some cells spontaneously reverted to an epithelial state after removing the cytokines, our results still indicated that the upregulation of GRHL2 promoted the EMT of keratinocytes.

### Impaired MET of epidermal cells promotes scar formation

**Fig. 6:**
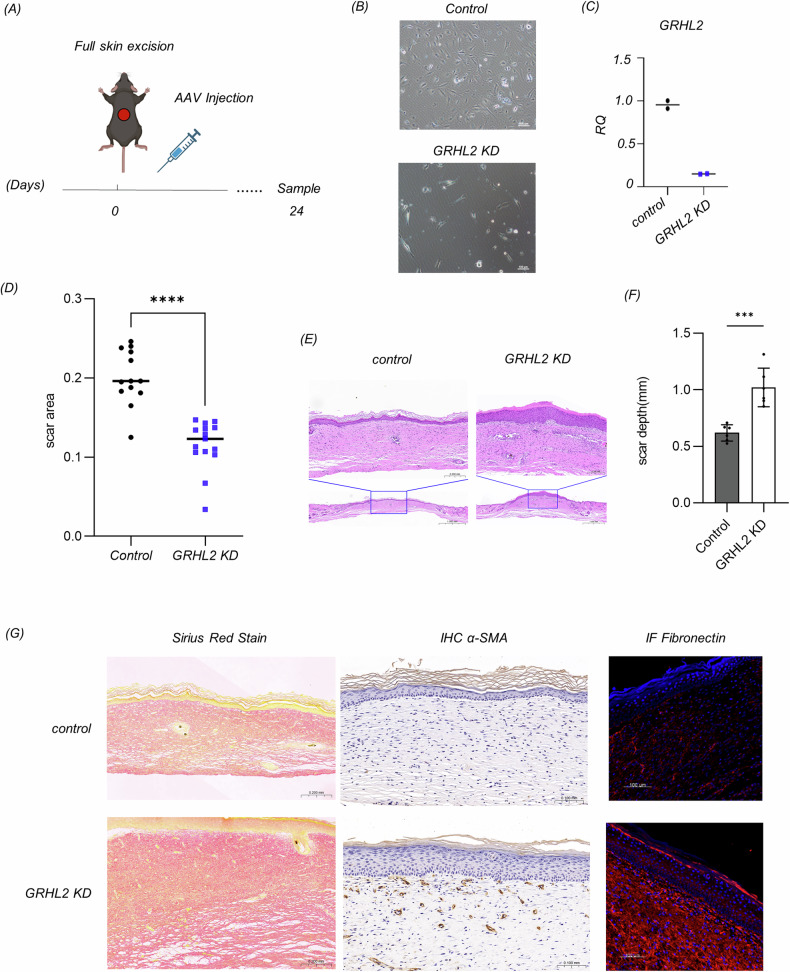
The persistent presence of EMT epidermal cells promoted scar tissue proliferation in mice. **A** Schematic diagram showing the exploration of impaired MET in epidermal cells during cutaneous wound healing. **B** Morphological changes of murine keratinocytes with GRHL2 knockdown. **C** The knockdown efficiency of GRHL2 shRNA in murine keratinocytes according to RT-qPCR, *n* = 2. **D** The scar surface area on day 24 of the control and GRHL2 KD group, determined based on photographs of the scars using ImageJ software. Control: Double-stranded small RNAs that do not target any known mammalian genes. The data are presented as means ± SD, *n* = 12, *p < 0.05, **p < 0.01, ***p < 0.001, ****p < 0.0001. **E** HE staining of mouse skin sections of the control and GRHL2 KD groups. Scale bar = 1000 μm (global view) or 200 μm (detailed view). **F** The depth of scar tissue of the control and GRHL2 KD group, determined based on microscopy images using ImageJ software. The data are presented as means ± SD, *n* = 6, *p < 0.05, **p < 0.01, ***p < 0.001, ****p < 0.0001. **G** Picrosirius red staining (Scale bar = 200 μm), immunohistochemistry for α-SMA (Scale bar = 100 μm), and immunofluorescence for fibronectin in the control and GRHL2 KD group (Scale bar = 1000 μm).

To investigate the effect of the failure of MET in epidermal cells on scar tissue formation, we chose AAV viral delivery of shRNA to interfere with the expression of GRHL2 in epidermal tissue at the wound edges (Fig. 6A). A vector encoding double-stranded small RNAs that do not target any known mammalian genes was used as a control. The knockdown efficiency of GRHL2 in mouse keratinocytes after transfection with the virus-delivered shRNA reached more than 80% (Fig. 6B, C).

Then, we delivered the AAV virus to the wound-adjacent skin via intradermal injection. Twenty-four days after the injection, we collected samples of the scar tissue. The area of the scar was diminished in the group of mice with GRHL2 knockdown (Fig. 6D). We hypothesize that this phenomenon may be attributed to an acceleration of re-epithelialization or activation of myofibroblasts by EMT epidermal cells through paracrine pathways. Myofibroblasts are known to facilitate wound contraction, thereby diminishing the scar area.

Based on the observation of HE-stained sections, scar tissue hyperplasia could be seen in the GRHL2 KD group, which was manifested as an increase of scar thickness (Fig. 6E, F). We observed the collagen density in the scar tissue by picrosirius red staining, wherein collagen fibers appeared red under bright-field microscopy. This showed that the collagen fiber density in the scar tissue was higher in the GRHL2 KD group than in the control group. Alpha-SMA is a commonly used marker for myofibroblasts, which is enriched in hypertrophic keloids and keloid scars [43]. Immunohistochemical results showed an increase of α-SMA-expressing myofibroblasts in the GRHL2 KD group. Additionally, fibronectin, known to be abundantly deposited in hypertrophic keloids and keloid scars [44], exhibited heightened levels in scar tissue, as indicated by our immunofluorescence staining results (Fig. 6G).

Taken together, these results demonstrate that the persistence of EMT epidermal cells promoted the formation of scar tissue.

### GRHL2 regulates EMT in keratinocytes through the miR-200s/Zeb1 axis

**Fig. 7:**
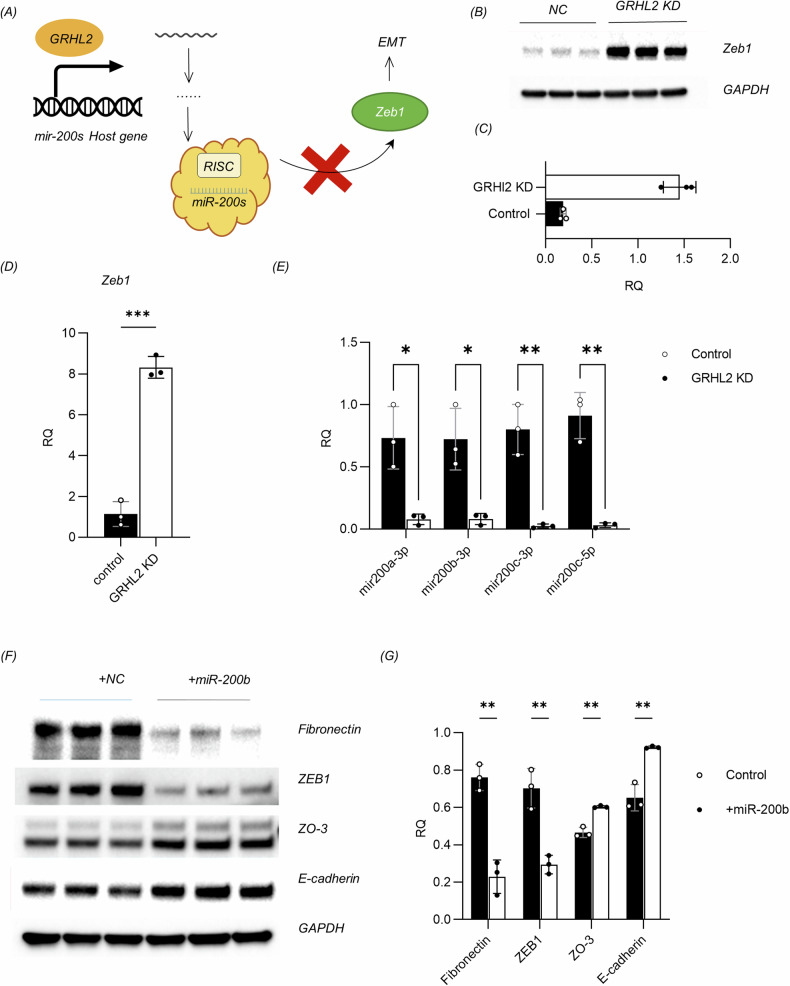
GRHL2 regulates keratinocytes through the Zeb1/miR-200s axis. **A** Schematic illustration of the regulation of EMT in keratinocytes by GRHL2 through the Zeb1/miR-200s axis. **B** Expression levels of EMT transcription factor Zeb1 in the control and GRHL2 KD keratinocytes according to western blot analysis. **C** Quantification of protein bands from the western blot in **B**. **D** Expression levels of EMT transcription factor Zeb1 according to RT-qPCR in the control and GRHL2 KD keratinocytes. The data are presented as means ± SD, *n* = 3. *p < 0.05, **p < 0.01, ***p < 0.001, ****p < 0.0001. **E** Expression levels of miR-200s, including miR-200a-3p, miR-200b-3p, miR-200c-3p, and miR-200c-5p, according to RT-qPCR in normal and GRHL2 KD keratinocytes. The data are presented as means ± SD, *n* = 3. *p < 0.05, **p < 0.01, ***p < 0.001, ****p < 0.0001. **F** Western blot analysis of the expression levels of fibronectin, Zeb1, ZO-3, and E-cadherin in GRHL2 KD keratinocytes transfected with miR-200b analogue or control small RNA. Control: Double-stranded small RNAs that do not target any known mammalian genes. **G** Quantification of protein bands from the western blot in **F**.

In order to investigate how GRHL2 regulates the EMT in keratinocytes during cutaneous wound healing, we refer to our transcriptomic analysis conducted on the GRHL2 KD keratinocyte cell line. The results showed that GRHL2 knockdown increased the transcriptional level of Zeb1, which is a master regulator of the EMT [45]. Studies in malignant tumors such as breast cancer and ovarian cancer have shown that the Zeb1/miR-200s axis is involved in the regulation of EMT by GRHL2 [46–49]. Zeb1 suppresses the miR-200 microRNA family, which in turn inhibits EMT by blocking Zeb1 translation, forming a negative feedback loop [50–52]. GRHL2 was found to stimulate the transcription of microRNA-200 family (miR-200s) host genes by binding directly to their promoters [46].Consequently, we hypothesized that GRHL2 interference could inhibit the expression of miR-200s and thus increase the mRNA and protein levels of Zeb1 in epidermal cells (Fig. 7A). The role of the Zeb1/miR-200s axis in regulating EMT-MET dynamics of keratinocytes during wound healing has not yet been investigated.

The western blot (Fig. 7B, C) and qPCR (Fig. 7D) results indicated that the expression of zeb1 was elevated in EMT cells following GRHL2 knockdown. At the same time, inhibition of GRHL2 expression drastically reduced the expression levels of small RNAs of the miR-200s family, including mir200a-3p, mir200b-3p, mir200c-3p and mir200c-5p (Fig. 7E).

To confirm these findings, we transfected EMT keratinocytes with miR-200b analogs. The results demonstrated that miR-200b transfection could reverse the spindle-like morphology of the cells (Supplementary Fig. 5). Additionally, it effectively reduced the expression of zeb1 and promoted the restoration of the epithelial state in EMT epidermal cells, which was manifested by decreased expression of fibronectin, as well as increased expression of E-cadherin and ZO-3 (Fig. 7F, G).

This indicates that GRHL2 at least partially regulates the EMT of keratinocytes by modulating the Zeb1/miR200s axis. Since GRHL2 is subject to post-transcriptional regulation, the delivery of miR-200s may more effectively reverse the mesenchymal phenotype of epithelial cells in vivo compared with mRNA transfection or introducing a DNA vector overexpressing GRHL2. Our results establish a theoretical basis for intervening with the EMT in epidermal cells and treating scarring through miR-200 delivery.

## Discussion

Currently, although scar tissue can be alleviated, achieving scarless cutaneous wound healing through medical intervention remains elusive. Therefore, it is imperative to focus on how the process of wound healing ceases in order to prevent excessive scar formation. Our experimental findings indicate that the persistence of EMT epidermal cells after wounds heal promotes the formation of scar tissue. In addition, GRHL2 is downregulated in keloids tissue, a type of abnormal hypertrophic scar. Hence, there is a pressing need to better understand the EMT of keratinocytes in aberrant tissues such as keloids.

Due to the unclear mechanisms underlying the persistent invasive growth of keloids, clinical interventions are often limited to passive defense measures such as surgical excision, local radiotherapy, and steroid injections, making treatment challenging. Therefore, there is an urgent need to study the mechanisms underlying the invasive growth of keloids. We have noticed that there are more reports on the EMT of keloid epidermal tissue than for normal hypertrophic scars. In our research, we have also confirmed a decrease of GRHL2 expression in EMT keratinocytes from keloids. Considering the elevated levels of TGF-β in keloid tissue [53], as well as its inhibitory effect on GRHL2 expression [54], we cannot conclusively determine that the loss of GRHL2 is the cause of keloid formation. However, studies of cancer and embryonic development have shown that the EMT can promote the invasion of cells into surrounding tissues, suggesting a potential association between epidermal EMT and keloid pathogenesis.

Moreover, the reduction of GRHL2 expression can affect the TGF-β, TNF-NFκB, and IL-6 signaling pathways of keratinocytes, thereby potentially influencing their interaction with the keloid microenvironment. In our study, we found that GRHL2 KD keratinocytes only exhibited an enhanced migratory ability compared to control cells when treated with TGF-β. This experimental result underscores the phenotypic impact of GRHL2 knockdown on keratinocyte responsiveness to TGF-β. Currently, we lack animal models for keloids, preventing us from exploring their potential treatment by inducing the MET in keratinocytes.

In studies on psoriasis, researchers have also identified keratinocytes undergoing the EMT [55, 56]. The phenotypic alteration of keratinocytes in psoriasis is believed to correlate with dysregulation of the IL-17 pathway. The secretion of IL-17 by Th17 cells may play an important role in the pathogenesis of psoriasis and has emerged as a potential therapeutic target [56, 57]. In this study, transcriptomic analysis of keratinocytes revealed that GRHL2 knockdown had a significant impact on both Th17 cell differentiation and the IL-17 signaling pathway (Supplementary figure 5), as indicated by KEGG pathway analysis. Moreover, a notable elevation of IL-17 levels was previously observed in keloids [58–60]. However, further research is needed to determine whether the decreased expression of GRHL2 in keloid keratinocytes can enhance the regulation of cellular EMT by IL-17.

Currently, it remains unknown if the loss of GRHL2 expression is associated with aggressive cutaneous squamous cell carcinoma or basal cell carcinoma. Notably, our results indicate that GRHL2 knockdown represses epidermal cell proliferation, and previous findings indicate that may lead to reduced telomerase activity in aging keratinocytes [23]. However, telomerase is re-activated to prevent telomere shortening in most advanced cancers. Furthermore, GRHL2 has been shown to promote the initiation of oral squamous cell carcinoma [61]. Therefore, GRHL2 may have a dual role in the development of non-melanoma skin cancers.

The pathological changes typical of scar tissue are mostly characterized by persistent inflammation in the dermis and the deposition of large amounts of collagen due to altered extracellular matrix metabolism in fibroblasts. Our study indicates that keratinocytes in the epidermis are not only influenced by the scar microenvironment and undergo phenotypic transformation, but in turn can also contribute to the scar microenvironment itself. EMT epithelial cells can alter the extracellular matrix metabolism of scar tissue by increasing the expression of matrix metalloproteinases such as MMP2 and MMP9 [34, 62]. They can also regulate the phenotype of other skin cells such as fibroblasts and macrophages by secreting IL-6 and other factors [36, 37]. Understanding how the abnormal phenotype of keratinocytes affects their communication with other components of the skin as well as the complex signaling pathways involved in regulating abnormal ECM deposition will lead to new treatments for scarring and keloids.

## Materials and methods

### Mice and wound healing model

The C57BL/6 J mice were aged 6 weeks at the beginning of experiments. They were bred and housed at the Tsinghua University Animal Facilities, with a 12 h light/dark cycle. The Animal Experiments Committee of Tsinghua University approved all of the experiments reported in this study (Approval No. 21-WQ1). Group allocation was randomized with no blinding.

The mice were anesthetized using an intraperitoneal injection of avertin (200 mg/kg). The dorsal surface was shaved with an electric clipper followed by a hair removal cream to remove the remaining hair. The skin as disinfected with iodine solution. A sterile 10-mm punch biopsy tool was used to outline the wounds on the dorsum. Then, the full-thickness wounds extending through the panniculus carnosus were made using iris scissors.

The adeno associated virus (AAV) vector was purchased from GeneChem Inc (Shanghai, China). Intradermal injections of approximately 20 μL per site were administered to deliver 1 × 10^13^ transducing units (TU) at 6 sites around the newly created wound in mice. The control group received the adeno associated virus expression vector with no inserted shRNA sequence in the same manner. Mice that died or had a significantly lower weight compared to others were excluded.

### Isolation of mouse epidermis

The mouse scar tissue was carefully removed wish scissors. To harvest the epidermis, the tissue was placed in a tube filled with PBS containing 200 IU penicillin and 200 mg/L streptomycin. Then, the skin tissue was cut thoroughly into small pieces and digested with Dispase II (2 mg/mL, Yeasen, China) in a tube or dish for more than 2 hours in a 37 °C water bath. After digestion, a new scalpel blade was used to scrape off the epidermis and hairs into the buffer. The total protein of the epidermis was extracted using Lysis Buffer for WB/IP Assays (Yeasen Biotechnology) according to the manufacturer’s instructions.

### HE and picrosirius red staining

The scar tissue was surgically removed and fixed in 4% paraformaldehyde, embedded in paraffin, and processed into 3μm slices. The sections were stained with hematoxylin and eosin (HE) or subjected to picrosirius red staining.

### Immunofluorescence staining and immunohistochemistry

For immunofluorescence staining, the tissue sections or coverslips with cells were blocked with QuickBlock™ Blocking Buffer for Immuno Staining (Beyotime, China) for 30 min at room temperature. Subsequently, the rat primary antibodies against mouse fibronectin (1:100, ab268020, Abcam, UK), E-cadherin (1:400, 70512, CST, USA), α-SMA (1:400, 19245, CST, USA), vimentin (1:200, 5741 CST, USA), ZO-1(1:100, ab221547, Abcam, UK), GRHL2 (1:1000, ab271023, Abcam, UK) and tenascin-C (YT5548, Immunoway Biotechnology, China) were incubated with the sample at 4 °C overnight. Then, an Alexa Fluor 555-labeled donkey anti-rabbit IgG secondary antibody (1:500, A0453, Beyotime, China) was used to stain the samples for 60 min at room temperature in the dark. Then, the sections were washed with TBS solution and incubated with the Alexa Fluor 488-conjugated rabbit monoclonal antibody against cytokeratin 14 (1:200, ab277277, Abcam, UK) at 4 °C overnight. The sections were mounted with mounting medium containing DAPI and covered with a glass slip. The stained sections were observed using a laser scanning confocal microscope (Zeiss, USA). The quantification of fluorescence and the intensity profile were performed using ImageJ.

### Cell lines and EMT/MET induction

Immortalized human HaCaT keratinocytes (Beina Chuanglian Biotechnology, China) were cultured in DMEM-high glucose medium supplemented with 10% FBS, 100 IU penicillin, and 100 mg/L streptomycin. HaCaT cells at passages 4-10 were used for further experiments.

Mouse epidermal keratinocytes were isolated as previously described [63]. After isolation, the keratinocytes were cultured in KGM Gold Keratinocyte Growth Basal Medium with supplements and growth factors from the Lonza™ KGM™ Gold Keratinocyte Growth Medium SingleQuots™ set according to the manufacturer’s instructions.

For EMT induction in vitro, we used recombinant TGF-β and EGF (both from PeproTech, USA). These proteins were dissolved according to the manufacturer’s instructions. For EMT or MET induction, cells were seeded at a density of 10^5^/cm^2^. After 12 hours, the culture medium was changed to serum-free (0% FBS) medium. After another 12 hours, the two factors were added to the medium, after which the culture medium was changed every two days. The control group was treated with serum-free culture medium. For MET induction, cells were washed with PBS before changing to the same medium but without the two factors.

All cells were maintained at 37 °C in a humidified incubator with 5% CO_2_.

### RNA isolation and RT-qPCR

Total RNA was extracted from cells using TRIzol reagent (Invitrogen, USA) according to manufacturer’s instructions. Then, 1 μg of the total RNA was reverse-transcribed using the StarScriptII First-strand cDNA Synthesis Mix with gDNA Remover (GenStar, China). The 2 × RealStar Green Power Mixture with ROXII (GenStar) was used to label the cDNA with fluorescent probes. Real-time quantitative polymerase chain reaction (RT-qPCR) was performed on a 7500 Fast Real-time PCR system (Thermo Fisher Scientific, USA), using the two-step RT-qPCR program parameters provided by the manufacturer. The primers are listed in Supplementary Table 1.

Cycle threshold (ΔΔCt) values were calculated by normalization to GAPDH, and the gene expression levels were compared using the 2^-ΔΔCt^ method.

For microRNAs, the total RNA (1 μg) was reverse-transcribed using the miRNA 1st Strand cDNA Synthesis Kit (by stem-loop) (Vazyme, China). The primers for reverse-transcription are listed in Supplementary Table 2. The miRNA Universal SYBR qPCR Master Mix (Vazyme, China) was used for quantification of microRNAs. The forward primer was specific, while the reverse primer for quantification was universal (named mir-qPCR-R in Supplementary Table 1).

### Total protein extraction and western blot analysis

Total protein of cells or animal tissues was extracted using Lysis Buffer for WB/IP Assays (Yeasen Biotechnology). Protein concentrations were determined using the BCA protein assay (Yeasen, China) according to the manufacturer’s instructions. An equivalent amount of total protein (20 μg) was subjected to sodium dodecyl sulfate–polyacrylamide gel electrophoresis (SDS–PAGE) on a 10% acrylamide gel and transferred to a PVDF membrane (0.45μm, Merck Millipore, Germany). The membranes were blocked for 30 min with 5% skimmed milk powder in T-BST at room temperature. The blocked membranes were individually incubated overnight at 4 °C with rabbit or mouse primary antibodies against fibronectin (1:1000, Ab268021, Abcam, US), ZO-1 (1:1000, 8193,CST, USA), E-cadherin (1:1000, 3195 T, CST, USA), α-SMA (1:1000, 19245, CST, USA), N-cadherin (1:20000, 66219-1-Ig, Proteintech, China), MMP2 (1: 1000, T57164, Abmart, China), ZO-3 (1:1000, ab181991, Abcam, UK), β-actin (1: 1000, GB5003-100, Servicebio, China), and GAPDH (1:1000, 5174, CST, USA), respectively. Then, the membranes were washed and incubated with secondary anti-rabbit antibodies (1:5000, CST, USA) or anti-mouse antibodies (1:5000, ZSB-BIO, China) for 1 h at 37 °C. The chemiluminescence signal was detected using Enlight buffer (Engreen, China). The optical densities of the bands were quantified using Image Lab software (Bio-Rad, USA) and normalized to GAPDH or β-actin as internal control. The densitometry of western blots was performed with ImageJ.

### Microscopy images

Brightfield images were recoded using an AMG EVOS Microscope at room temperature. Cells were imaged using a 10× objective.

### Cell viability assay

Cells were seeded into 96-well plates. Following culture for varying durations (12 h and 72 h), the cells were treated with 10 μL/well of the CCK-8 solution (Cell Counting Kit 8, Beijing BioDee Biotechnology, China) and incubated for 2 hours at 37 °C. Absorbance at 450 nm was determined using a conventional microplate reader.

### Lentivirus production and infection of cells

The lentiviral expressing vector carrying the shRNA targeting GRHL2 was obtained from the shRNA library of Tsinghua University (ID: TRCN0000015810). The sequence for shRNA was: CCGGGCTGAAGATTTCACACCAGTTCTCGAGAACTGGTGTGAAATCTTCAGCTTTTT. The control vector was the MISSIONO shRNA Plasmid DNA Control Vector (Catalog Number SHC002). For lentivirus production, HEK293T cells were transfected with the lentivirus expression plasmid, lentivirus packing plasmid PSPAX2 and lentivirus envelope expression plasmid PMD2.G at a mass ration of 2:3:1. The supernatant containing the lentivirus particles was harvested at 48 hours post transfection, filtered through 0.45μm pore-size membrane and used to infect 20% confluent HaCaT cells together with 10 mg/L polybrene (Beyotime, China). The culture medium was changed 12-16 hours after transfection. Following 72 hours after transfection, selection for positive cells was conducted with puromycin (2 mg/L, Solarbio) in the regular growth medium for 3 days. A monoclonal cell line was generated by limiting dilution. Then, RT-qPCR was used to measure the knockdown efficiency.

The RNA samples for RNA-seq were extracted using TRIzol reagent (Invitrogen, USA) according to manufacturer’s instructions. The sequencing and analysis were conducted by GENEWIZ Inc. (Suzhou, China).

### Transfection with miRNAs

The miRNAs were purchased from GenePharma Corporation (Shanghai, China). The sequences were as follows: siNC: 5’-UUCUCCGAACGUGUCACGUTT-3’; miR-200b analog: 5’-UAAUACUGCCUGGUAAUGAUGA3’. Cells were seeded into 12-well-plates at a density of 1.5 × 10^5^/cm^2^. Following 12 hours after seeding, the cells were transfected with the negative control RNA molecule (siNC) or miR-200b analog using Lipofectamine 3000 (Thermo Fisher, USA) according to the manufacturer’s instructions. The final concentration of the siRNA solution was 50 nM.

### Patients and sample collection

The research complied with the Declaration of Helsinki and it was approved by the ethics committee of Beijing Tsinghua Changgung Hospital. Total of three keloid tissue specimens were obtained from patients undergoing scar excision therapy at Beijing Tsinghua Changgung Hospital in 2023. All participants provided written informed consent.

### Statistical analysis

The sample size was determined according to the typical sample size used in the corresponding experimental method. The results are shown as means ± standard errors of the mean. GraphPad Prism 9.5 (GraphPad Software Inc., USA) was used for data processing and graphing. The statistical significance of differences was assessed using two-tailed Student’s *t*-test. Differences with *P* < 0.05 were considered statistically significant (*P < 0.05, **P < 0.01, ***P < 0.001, ****P < 0.0001).

## Supplementary information

Supplementary figures & tables

Original western blot

## Data Availability

The datasets generated during and/or analyzed during the current study are available from the corresponding author on reasonable request.
