# Supplementary material for: GRHL2 regulates keratinocyte EMT-MET dynamics and scar formation during cutaneous wound healing
Source: Cell Death Dis. 2024 Oct 14;15(10):748. doi: 10.1038/s41419-024-07121-7 (PMC11473813; doi:10.1038/s41419-024-07121-7)
Supplement: Supplementary file 1 — Supplementary figures & tables [file 41419_2024_7121_MOESM1_ESM.docx]

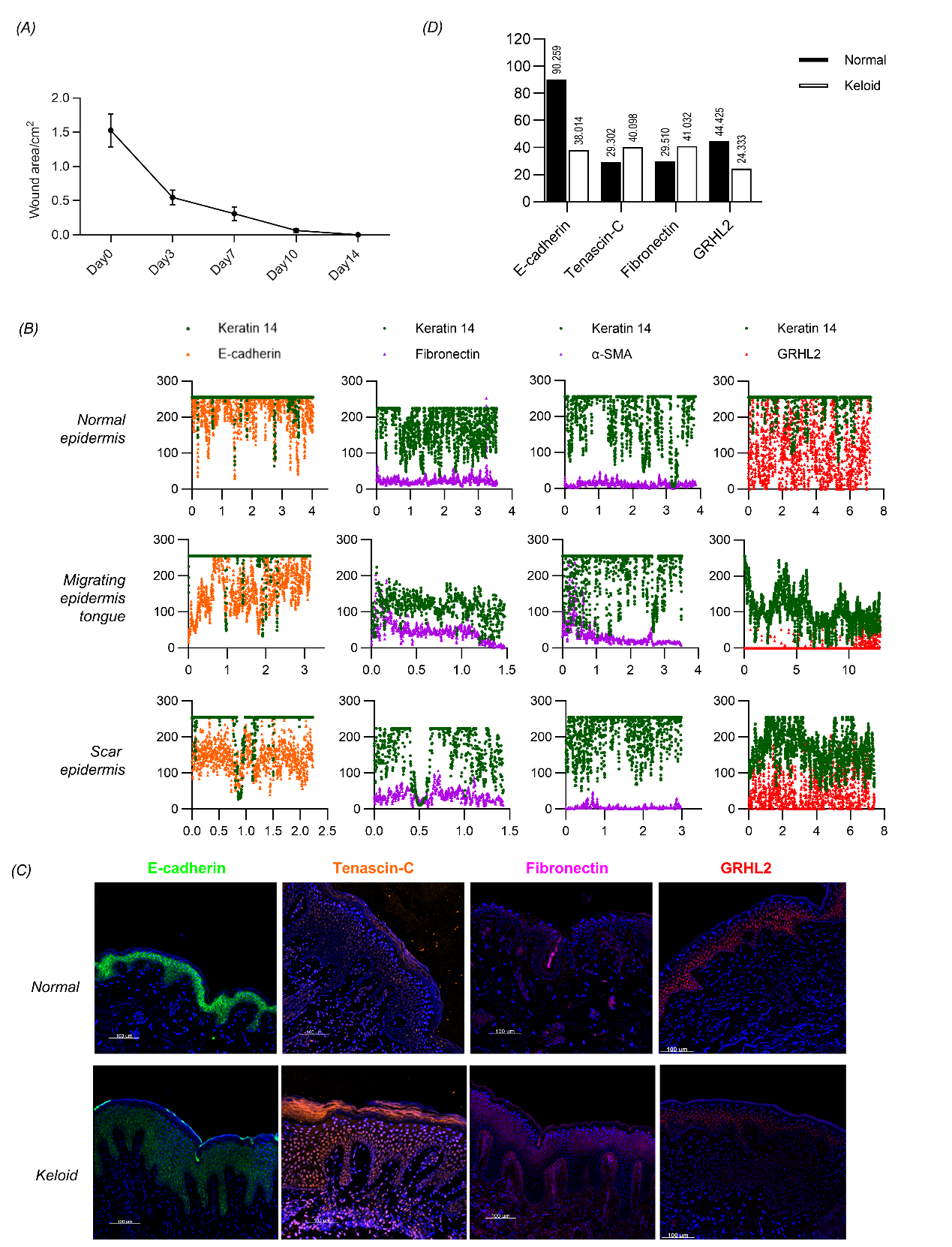


**Supplementary figure 1** (A) Kinetics of wound healing in mice with 1 cm-diameter full thickness wounds on the dorsum. Each point represents the mean of the area of the wound size ± SD of wounds harvested from five mice. (B) The intensity profile of fluorescence along the epidermis for immunofluorescence analysis in Figure 1D. The X- axis represent distance while the Y- axis represent fluorescence intensity. In the epidermis at the wound edge, the zero point on the X-axis corresponds to the wound edge. In the epidermis at normal and scar skin, the zero point on the X-axis corresponds to the left or right point of the epidermis. (C) Immunofluorescence analysis of GRHL2, E-cadherin, fibronectin, and tenascin-C in normal skin and keloid tissue. Blue indicates cell nuclei. The scale bars represent 100 μm. Epithelial marker: E-cadherin; Mesenchymal markers: fibronectin, tenascin-C. (D) Quantification of fluorescence intensity in the epidermis area based on the immunofluorescence analysis in Supplementary Figure 1C.


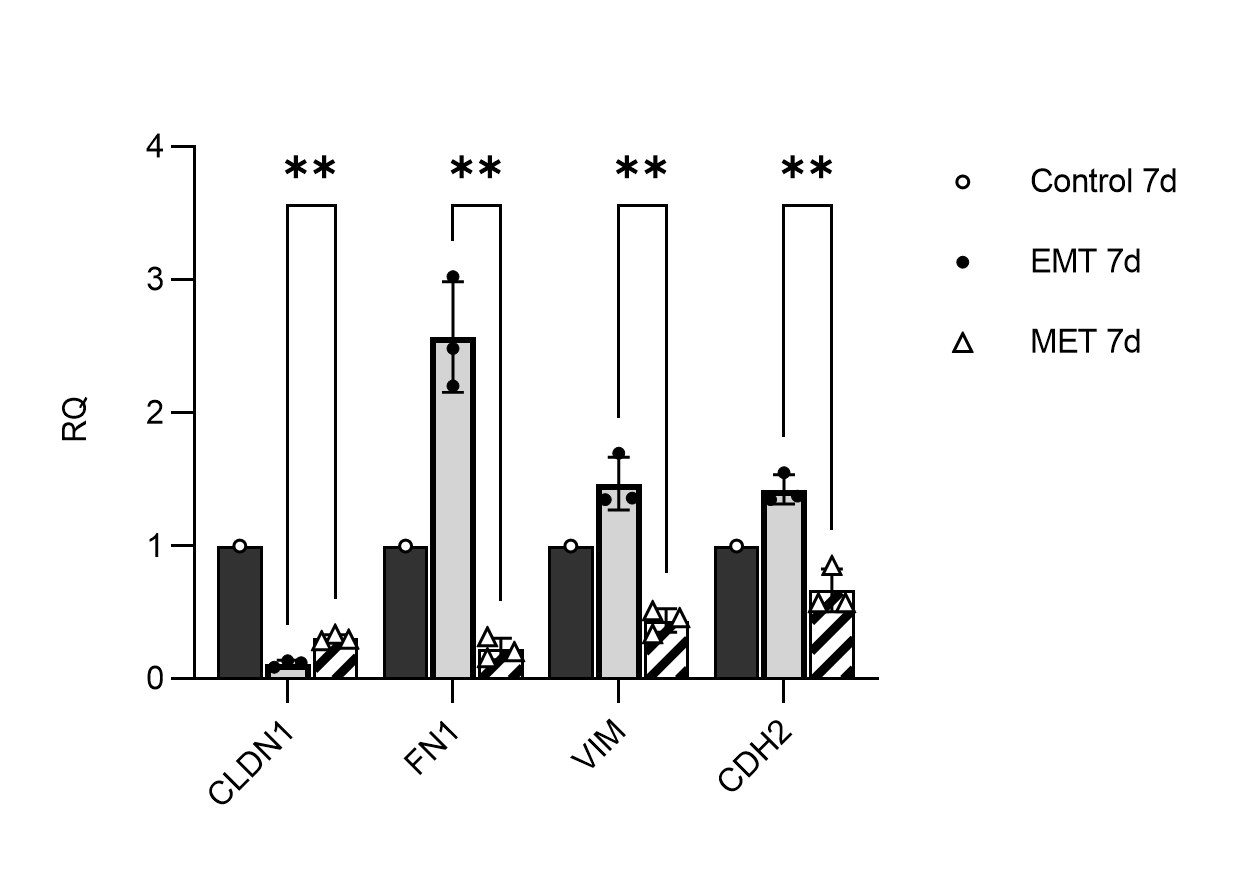


**Supplementary figure 2** Expression levels of epithelial and mesenchymal markers in the control, EMT and MET groups, as determined by RT-qPCR. The data are presented as means ± SD, *p < 0.05, **p < 0.01, ***p < 0.001, ****p < 0.0001, n = 3.


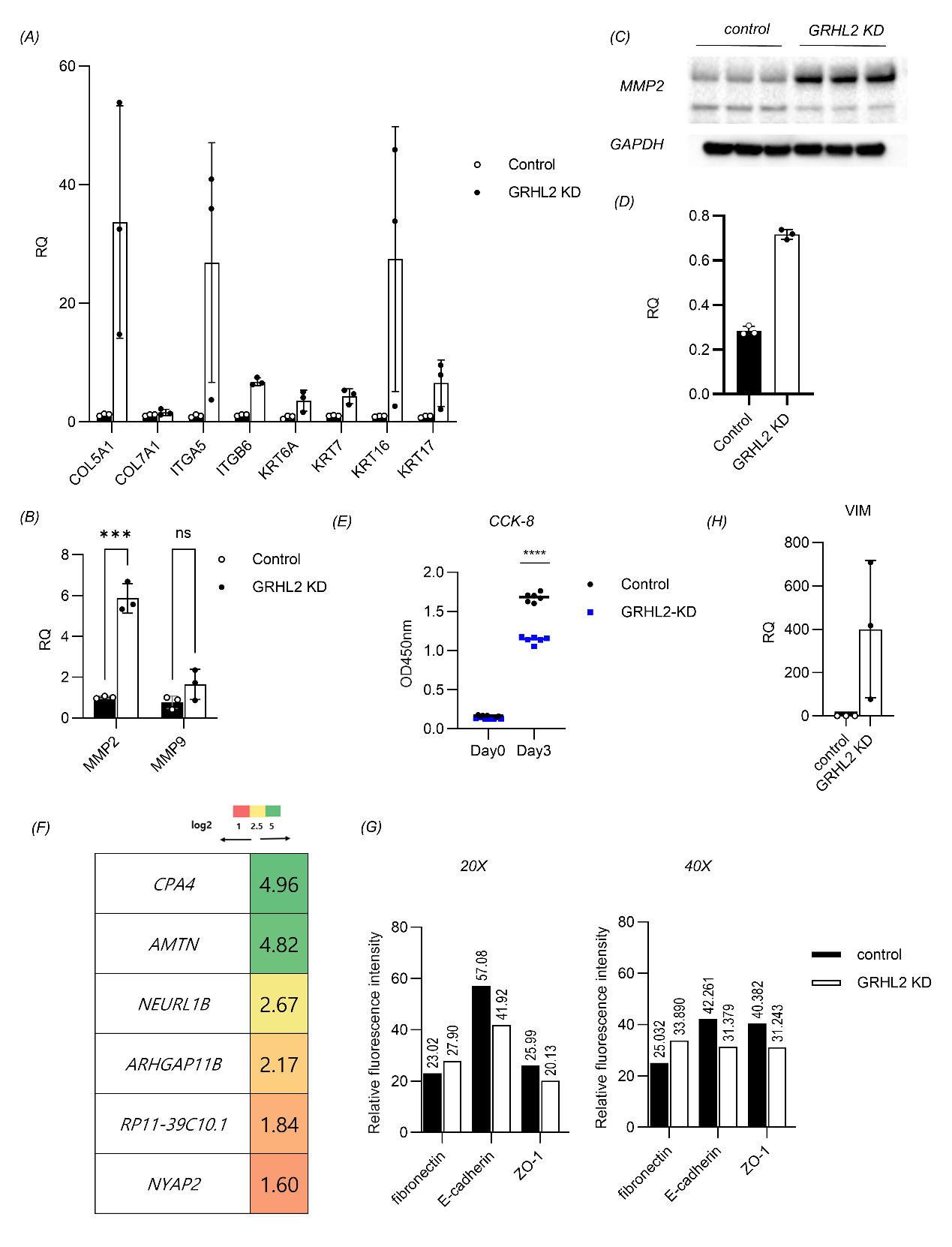


**Supplementary figure 3** (A) Expression levels of representative genes coding collagens, keratins and cytokines according to RT-qPCR in normal and GRHL2 KD keratinocytes. The data are presented as means ± SD, n = 3. (B) Expression levels of matrix metalloproteinases MMP2 and MMP9 according to RT-qPCR in normal and GRHL2 KD keratinocytes. The data are presented as means ± SD, n = 3, *p < 0.05, **p < 0.01, ***p < 0.001, ****p < 0.0001. (C) Expression levels of MMP2 according to western blot analysis in normal and GRHL2 KD keratinocytes. (D) Quantification of protein bands from the western blot in Supplementary figure 3C. (E) CCK-8 assay for determining the proliferative capacity of control and GRHL2 KD keratinocytes. *p < 0.05, **p < 0.01, ***p < 0.001, ****p < 0.0001, n = 6. (G) Quantification of fluorescence intensity according to immunofluorescence analysis in Figure.4E. Cells with activated Vimentin expression are very rare, so we did not perform quantification. (F) Transcriptomic analysis of cell-cycle related genes (GRHL2 KD cells compared with control keratinocytes). (H) Expression of vimentin (VIM) according to RT-qPCR in normal and GRHL2 KD keratinocytes. The data are presented as means ± SD, n = 3.

**
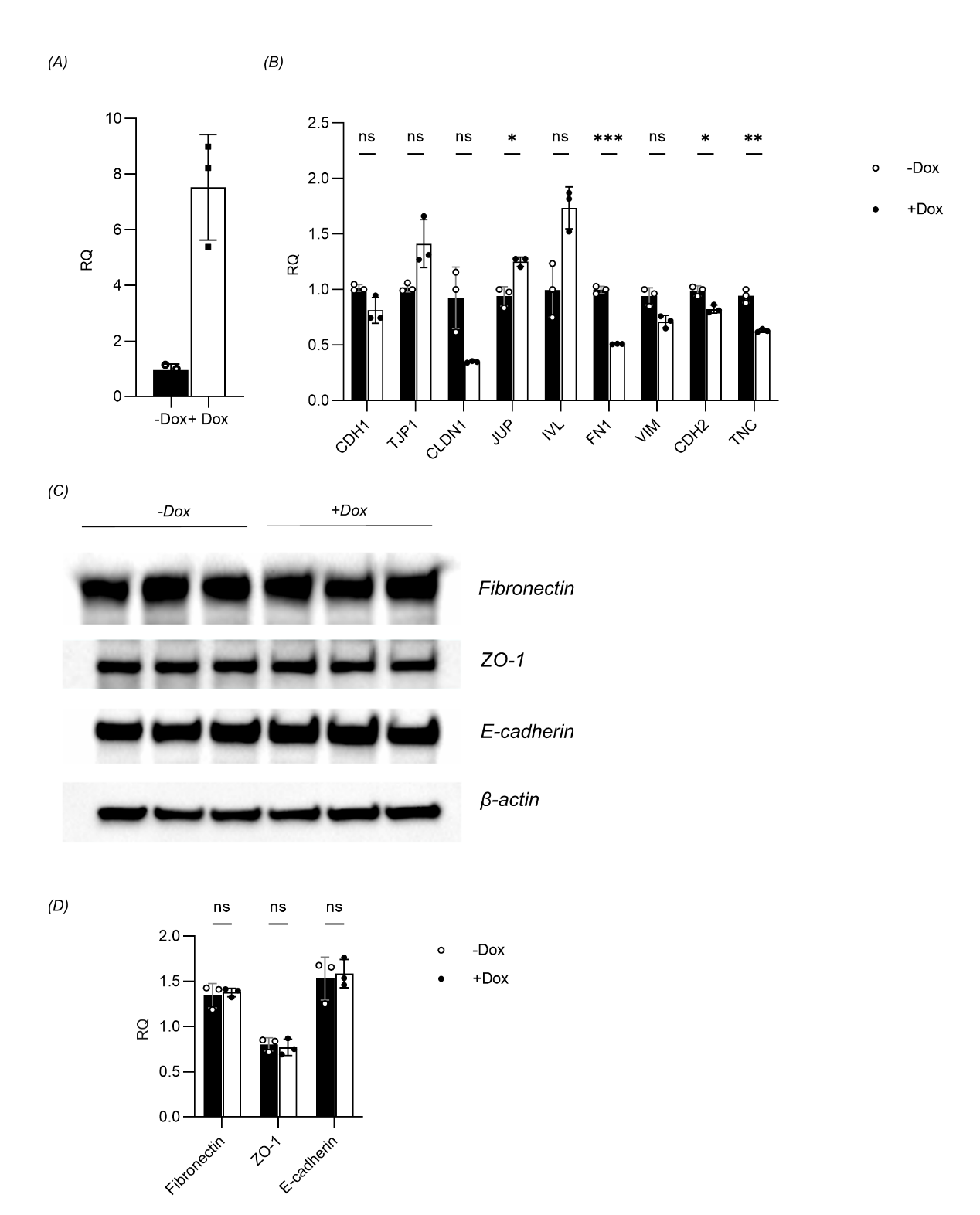
**

**Supplementary figure 4** (A) Overexpression efficiency of GRHL2 in EMT keratinocytes according to RT-qPCR. (B) Expression levels of EMT-related markers according to RT-qPCR in EMT keratinocytes treated with doxycycline (Dox) or left untreated. Error bars represent standard deviations, n = 3. *p < 0.05, **p < 0.01, ***p < 0.001, ****p < 0.0001. CDH1 (E-cadherin), TJP1 (ZO-1), CLDN1 (Ccaudin-1), JUP (junction plakoglobin), IVL (involucrin), FN1 (fibronectin), VIM (vimentin), CDH2 (N-cadherin), TNC (tenascin-C). (C) Expression levels of fibronectin, ZO-1 and E-cadherin according to western blot analysis in EMT keratinocytes treated with Dox or left untreated. (D) Quantification of protein bands from the western blot in Supplementary figure 4C.


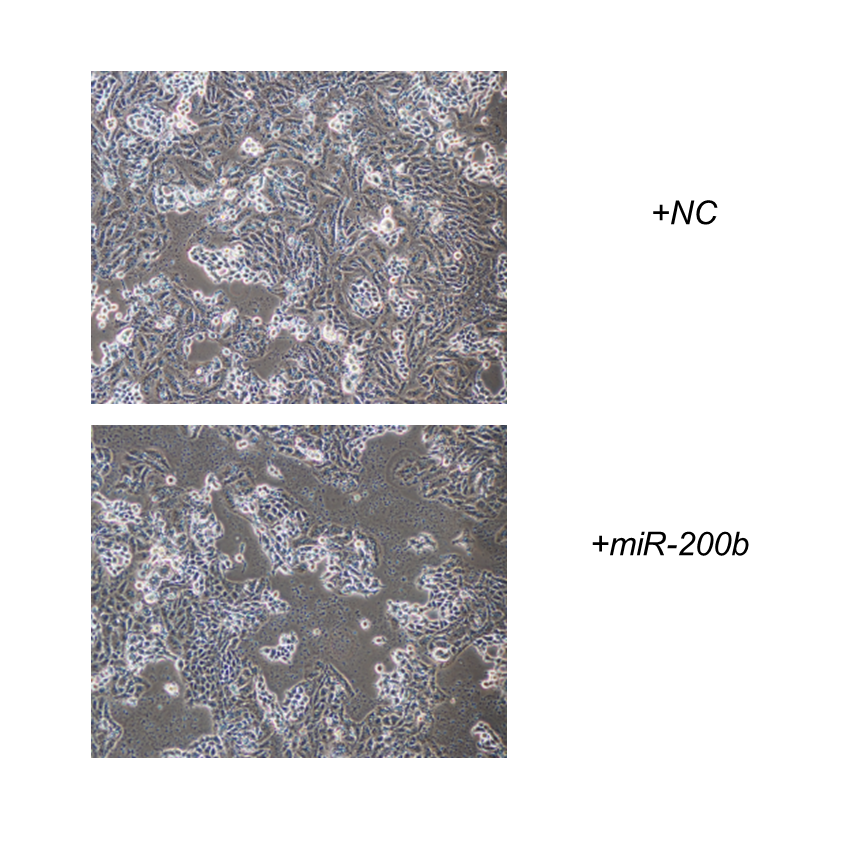


**Supplementary figure 5** Morphological changes of EMT keratinocytes transfected with miR-200b analogue.


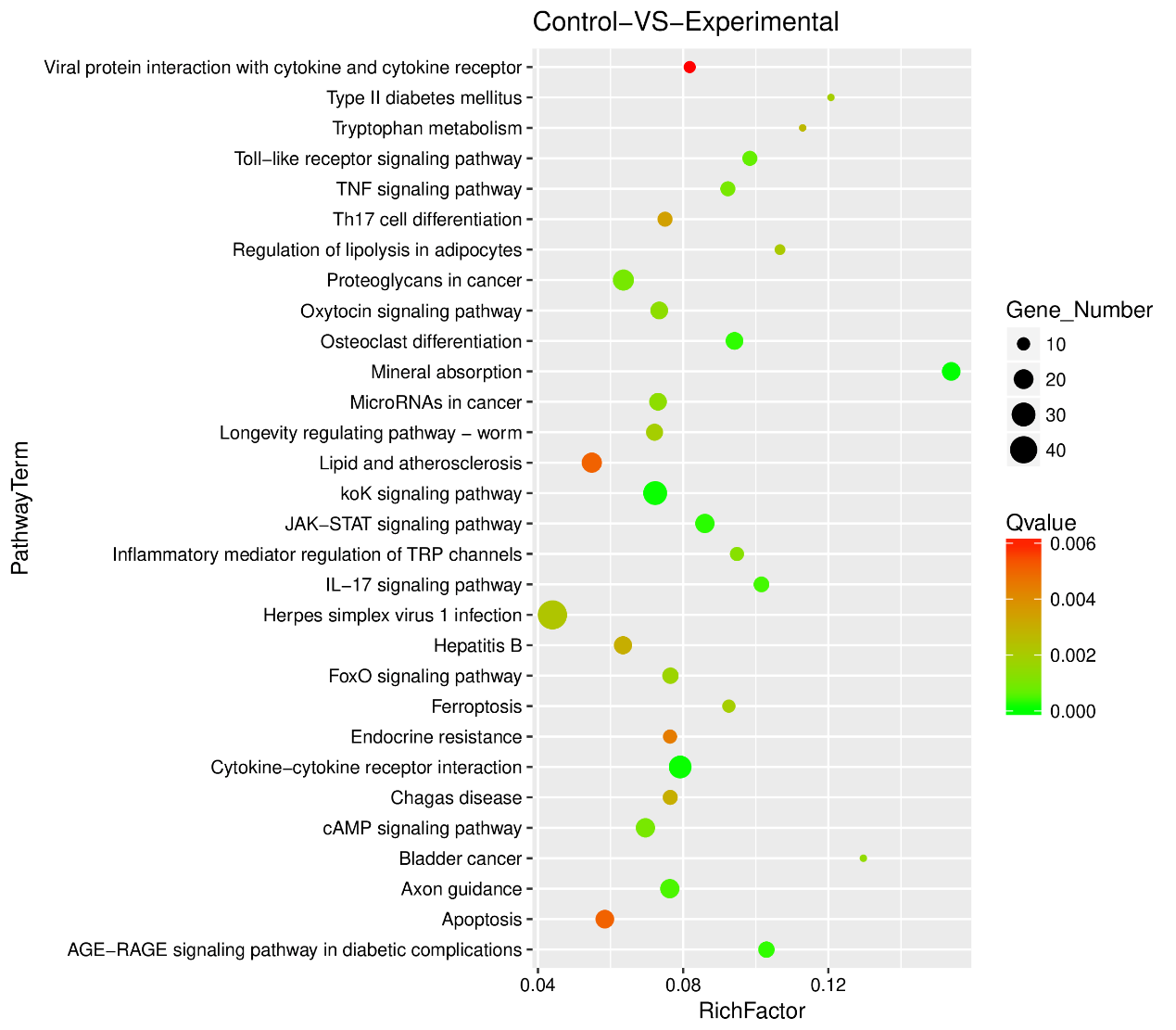


**Supplementary figure 6** KEGG pathway enrichment analysis of keratinocytes with GRHL2 knockdown compared to the control group.

**Supplementary Tables：**

**Supplementary table 1 RT-qPCR primer sequences**

| **RT-qPCR primer sequences** | |
| --- | --- |
| H-CDH1 F | ATTTTTCCCTCGACACCCGAT |
| H-CDH1 R | TCCCAGGCGTAGACCAAGA |
| H-VIM F | GACGCCATCAACACCGAGTT |
| H-VIM R | CTTTGTCGTTGGTTAGCTGGT |
| H-CDH2 F | AGCCAACCTTAACTGAGGAGT |
| H-CDH2 R | GGCAAGTTGATTGGAGGGATG |
| H-TJP1 F | ACCAGTAAGTCGTCCTGATCC |
| H-TJP1 R | TCGGCCAAATCTTCTCACTCC |
| H-DSP F | CAGCCCTGTGATGCTTACCAG |
| H-DSP R | ACTCTGACAAGTGTAGCCTCC |
| H-JUP F | TCTCCAACCTGACATGCAACA |
| H-JUP R | CATAGTTGAGACGCACAGAGTTC |
| H-GRHL2 F | GAAAACCGAGTGCAAGTCCTA |
| H-GRHL2 R | GGGCCATGAAAACTGGTGTG |
| H-FN1 F | CGGTGGCTGTCAGTCAAAG |
| H-FN1 R | AAACCTCGGCTTCCTCCATAA |
| H ZEB1 F | GATGATGAATGCGAGTCAGATGC |
| H ZEB2 R | ACAGCAGTGTCTTGTTGTTGT |
| H-GAPDH-F | ACAACTTTGGTATCGTGGAAGG |
| H-GAPDH-R | GCCATCACGCCACAGTTTC |
| H-beta-actin-F | CATGTACGTTGCTATCCAGGC |
| H-beta-actin-R | CTCCTTAATGTCACGCACGAT |
| H-IVL-F | GACTGCTGTAAAGGGACTGCC |
| H-IVL-R | CATTCCCAGTTGCTCATCTCTC |
| H-MMP2-F | TACAGGATCATTGGCTACACACC |
| H-MMP2-R | GGTCACATCGCTCCAGACT |
| H-MMP9-F | TGTACCGCTATGGTTACACTCG |
| H-MMP9-R | GGCAGGGACAGTTGCTTCT |
| H-COL5A1-F | GCCCGGATGTCGCTTACAG |
| H-COL5A1-R | AAATGCAGACGCAGGGTACAG |
| H-COL7A1-F | TTACGCCGCTGACATTGTGTT |
| H-COL7A1-R | ACCAGCCCTTCGAGAAAGC |
| H-TGFB2-F | CCATCCCGCCCACTTTCTAC |
| H-TGFB2-R | AGCTCAATCCGTTGTTCAGGC |
| H-TNFa-F | CCTCTCTCTAATCAGCCCTCTG |
| H-TNFa-R | GAGGACCTGGGAGTAGATGAG |
| H-IL6-F | CCTGAACCTTCCAAAGATGGC |
| H-IL6-R | TTCACCAGGCAAGTCTCCTCA |
| H-PDGFB-F | CTCGATCCGCTCCTTTGATGA |
| H-PDGFB-R | CGTTGGTGCGGTCTATGAG |
| H-ITGB6-F | GAGGACTACCCGGTGGATTTG |
| H-ITGB6-R | TCCTTTATTGTGTTGAGGTCGTC |
| H-ITGA5-F | GGCTTCAACTTAGACGCGGAG |
| H-ITGA5-R | TGGCTGGTATTAGCCTTGGGT |
| H-KRT7-F | GAGGAGAGCGAGCAGATCAAG |
| H-KRT7-R | CCCGAAGGCCAGCAATCTG |
| H-KRT16-F | GACCGGCGGAGATGTGAAC |
| H-KRT16-R | CTGCTCGTACTGGTCACGC |
| H-KRT17-F | CCAGATCCAGGGGCTGATTG |
| H-KRT17-R | CCACAATGGTACGCACCTGA |
| H-TGFB1-F | TACCTGAACCCGTGTTGCTCTC |
| H-TGFB1-R | GTTGCTGAGGTATCGCCAGGAA |
| H-CLDN1-F | CCTCCTGGGAGTGATAGCAAT |
| H-CLDN1-R | GGCAACTAAAATAGCCAGACCT |
| H-TNC-F | TCCCAGTGTTCGGTGGATCT |
| H-TNC-R | TTGATGCGATGTGTGAAGACA |
| H-IVL-F | GACTGCTGTAAAGGGACTGCC |
| H-IVL-R | CATTCCCAGTTGCTCATCTCTC |
| mir-qPCR-R | AGTGCAGGGTCCGAGGTATT |
| mir200a-3p-F | GCGCATCTTACCGGACAGT |
| mir200b-3p-F | GCGCATCTTACTGGGCAGC |
| mir200c-3p-F | GCGCGTCTTACCCAGCAGT |
| mir200c-5p-F | CGCGTAATACTGCCGGGTAAT |

**Supplementary table 2 Reverse-transcription primer sequences**

| **Reverse-transcription primer sequences** | |
| --- | --- |
| RT-mir200a-3p | GTCGTATCCAGTGCAGGGTCCGAGGTATTCGCACTGGATACGACTCCAGC |
| RT-mir200b-3p | GTCGTATCCAGTGCAGGGTCCGAGGTATTCGCACTGGATACGACTCCAAT |
| RT-mir200c-3p | GTCGTATCCAGTGCAGGGTCCGAGGTATTCGCACTGGATACGACCCAAAC |
| RT-mir200c-5p | GTCGTATCCAGTGCAGGGTCCGAGGTATTCGCACTGGATACGACTCCATC |
