# Supplementary material for: GRHL2 regulates keratinocyte EMT-MET dynamics and scar formation during cutaneous wound healing
Source: Cell Death Dis. 2024 Oct 14;15(10):748. doi: 10.1038/s41419-024-07121-7 (PMC11473813; doi:10.1038/s41419-024-07121-7)
Supplement: Supplementary file 2 — Original western blot [file 41419_2024_7121_MOESM2_ESM.pdf]

In our study, several EMT related markers was analyzed at the same time to evaluate whether the EMT or MET occurred in keratinocytes. During antibody incubation, we cut the membrane into strips while the parts could be spliced together. And we cropped along the markers. When detecting the chemiluminescence, we spliced the bands together and imaged them together to prove that they are from the same experiment. We will provide the original files, unedited western blot image, and photos of the strips spliced during the exposure as well as uncropped membrane with colorful protein markers.

Figure.1B

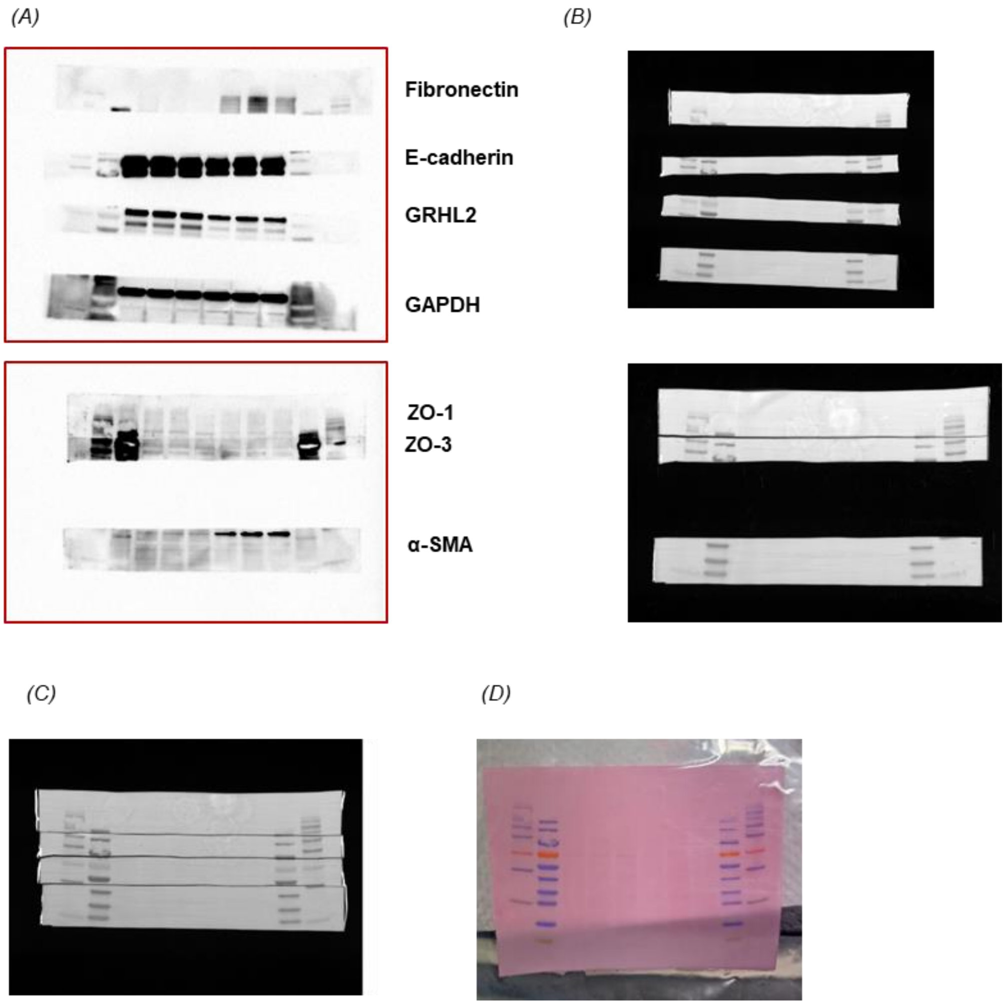

(A) Unedited western blot image; (B) Photos of the strips during the exposure; (C) Spliced bands; (D) Uncropped membrane.

Figure.2D

(A)

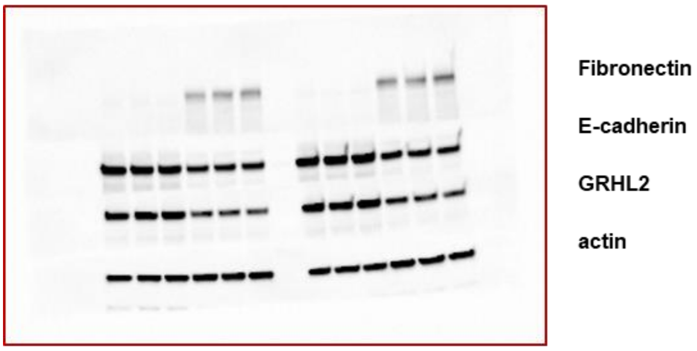

(B)

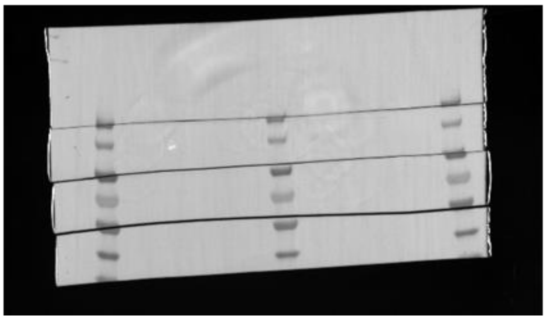

(C)

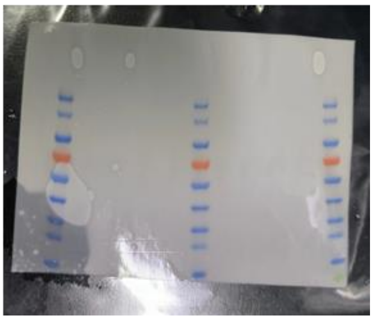

(A) Unedited western blot image; (B) Photos of the strips spliced during the exposure; (C) Uncropped membrane.

Figure.2F

(A)

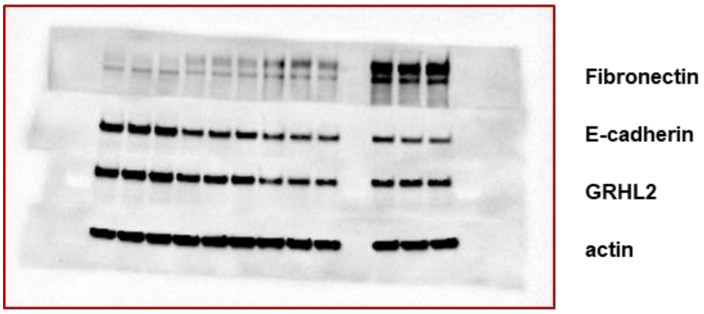

(B)

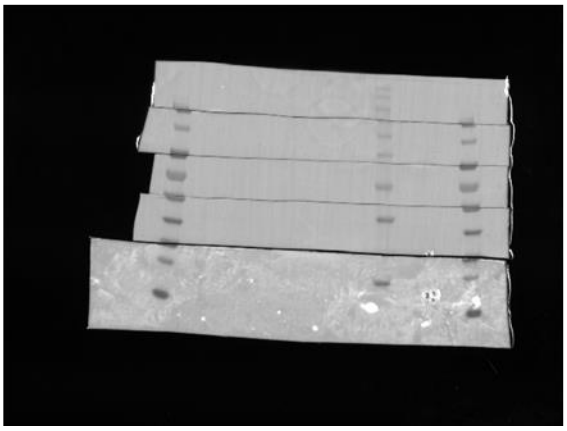

(C)

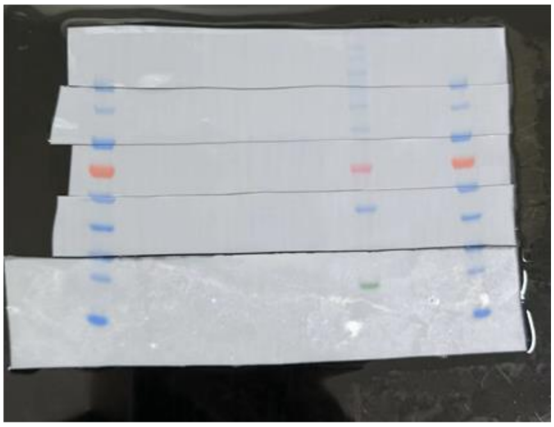

(A) Unedited western blot image; (B) Photos of the strips spliced during the exposure; (C) Colorful photos of the strips spliced during the exposure;

Figure.4B (Gel-1)

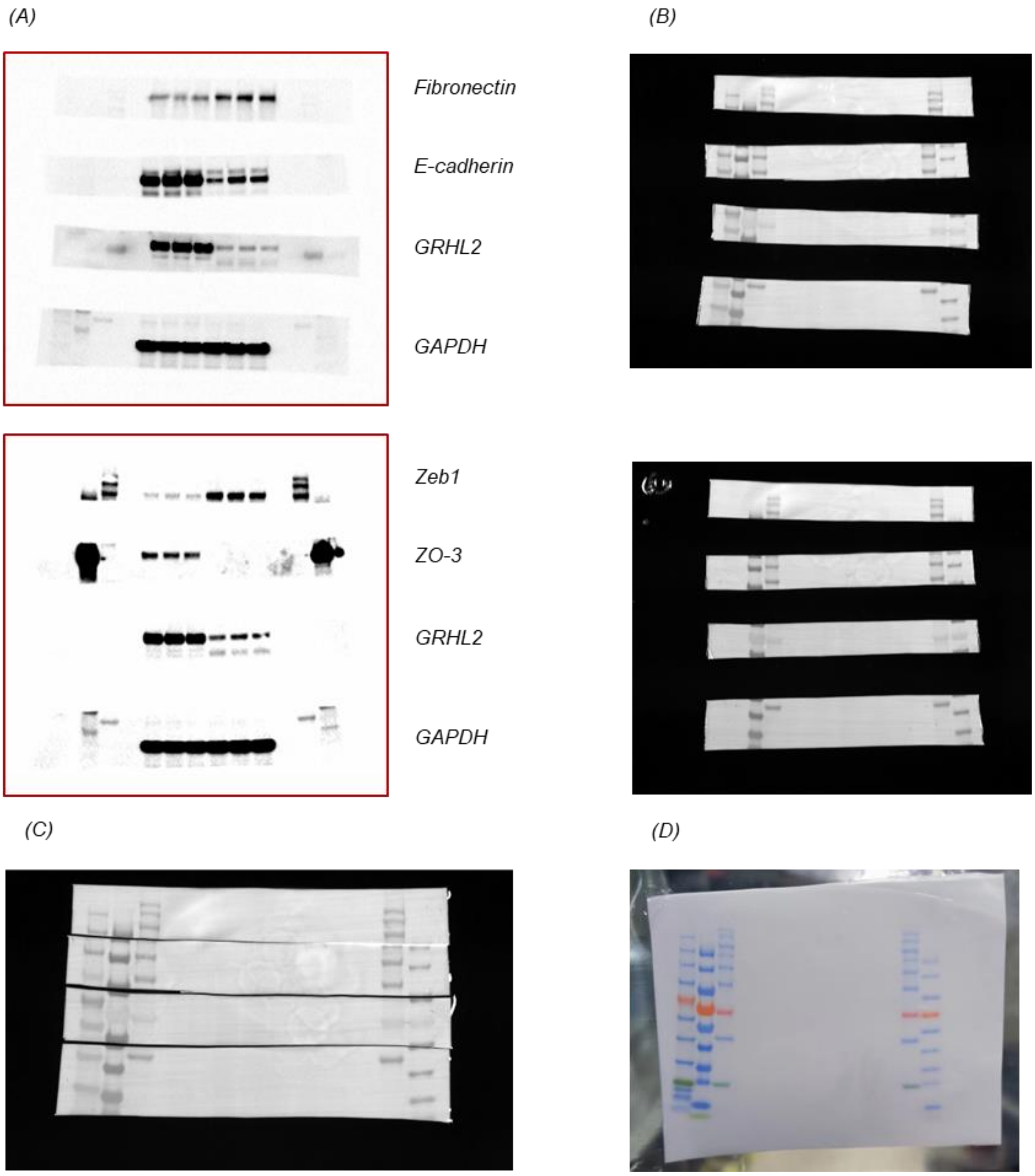

(A) Unedited western blot image; (B) Photos of the strips during the exposure; (C) Spliced bands; (D) Uncropped membrane.

Figure.4B (Gel-2)

(A)

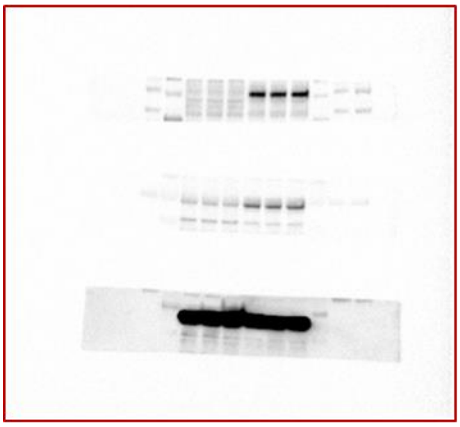

(B)

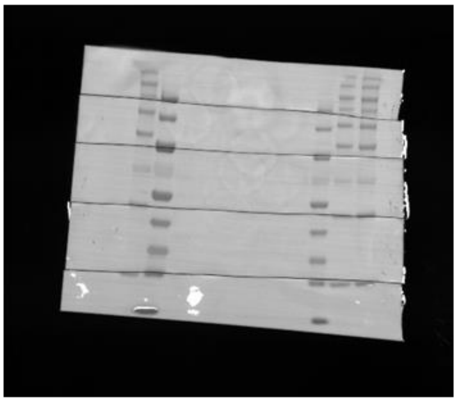

(C)

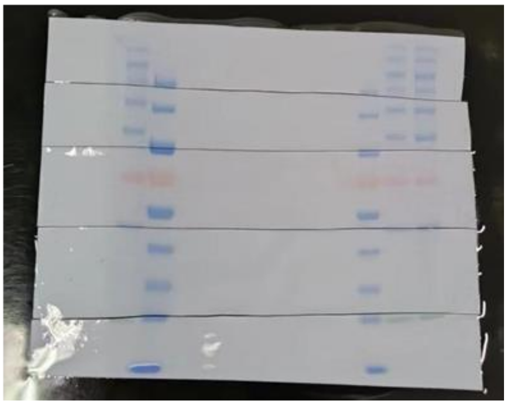

(A) Unedited western blot image; (B) Photos of the strips spliced during the exposure; (C) Colorful photos of the strips spliced during the exposure;

Figure.5E

(A)

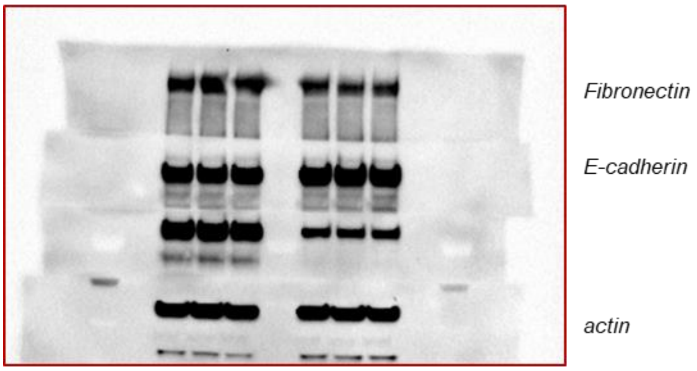

(B)

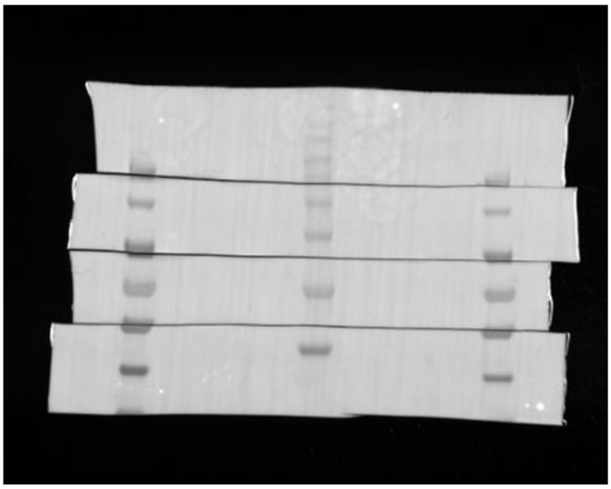

(C)

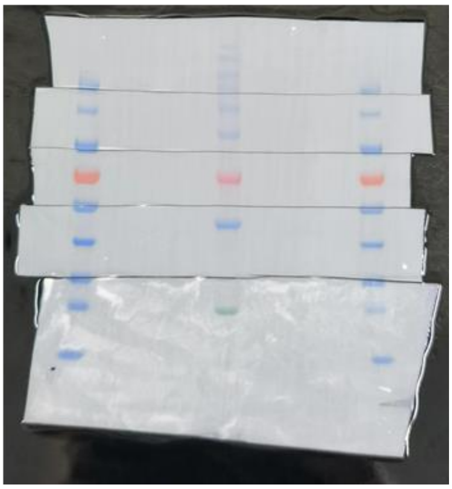

(A) Unedited western blot image; (B) Photos of the strips spliced during the exposure; (C) Colorful photos of the strips spliced during the exposure;

Figure.7B

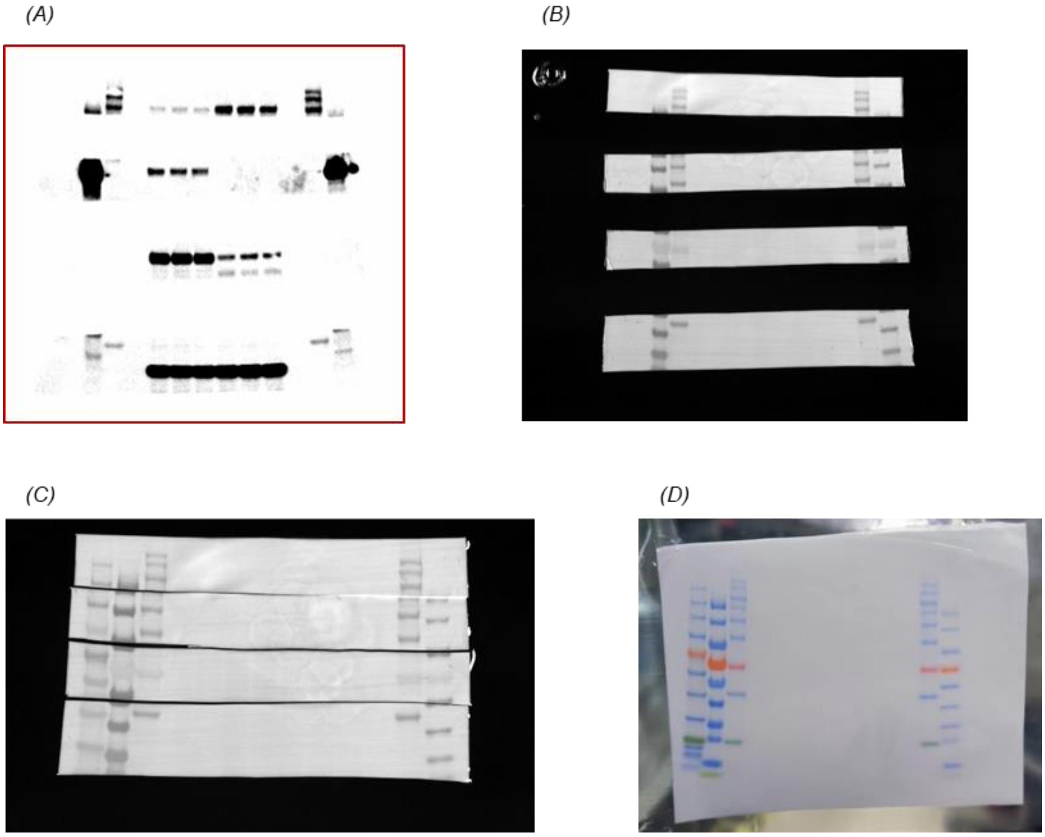

(A) Unedited western blot image; (B) Photos of the strips during the exposure; (C) Spliced bands; (D) Uncropped membrane.

Figure.7F

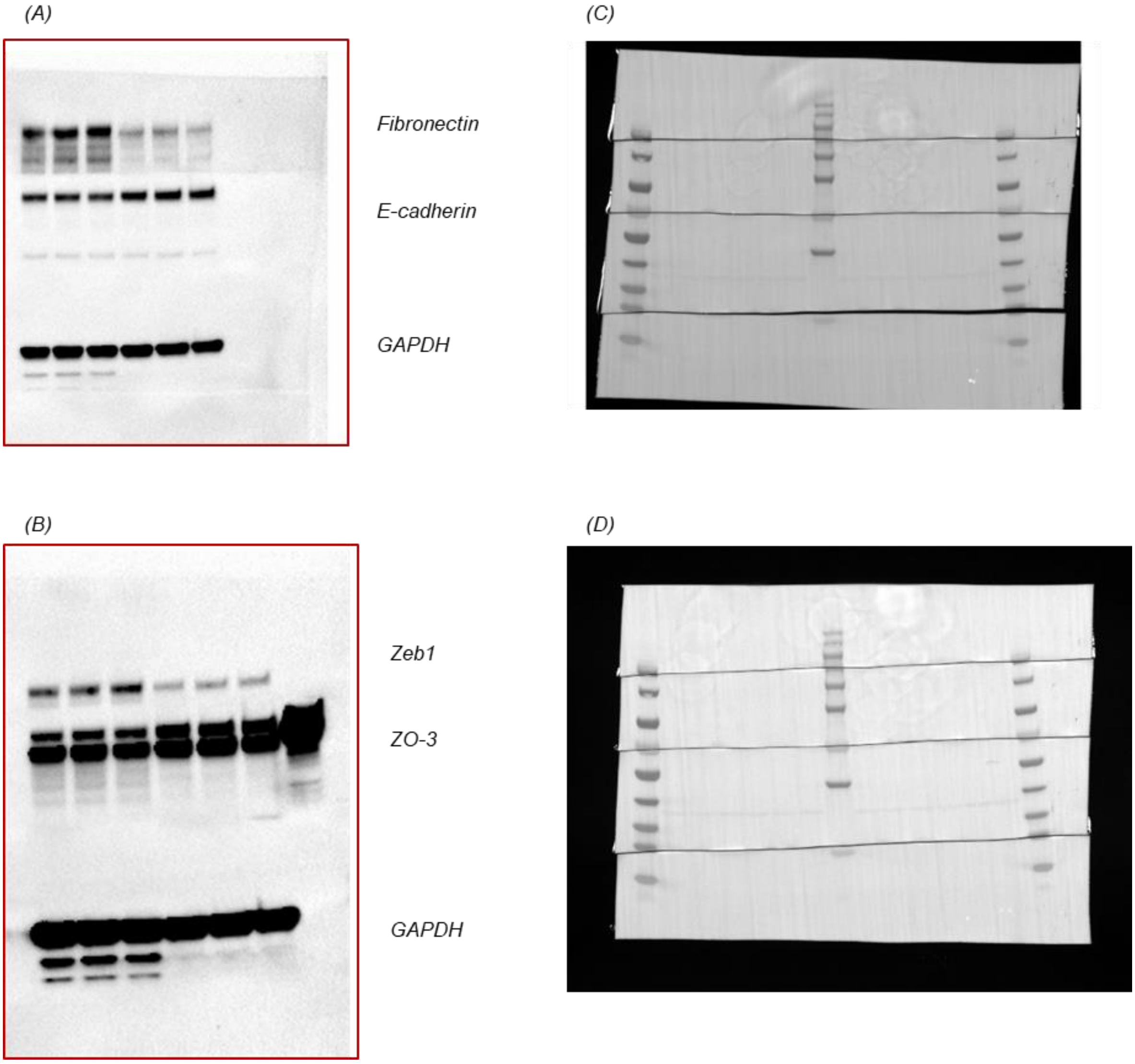

(A) Unedited western blot image; (B) Unedited western blot image; (C) Photos of the strips during the exposure; (D) Photos of the strips during the exposure.
